# Recovery of Precious Metals: A Promising Process Using Supercritical Carbon Dioxide and CO_2_-Soluble Complexing Polymers for Palladium Extraction from Supported Catalysts

**DOI:** 10.3390/molecules28176342

**Published:** 2023-08-30

**Authors:** Andrea Ruiu, W. S. Jennifer Li, Marin Senila, Cécile Bouilhac, Dominique Foix, Bernhard Bauer-Siebenlist, Karine Seaudeau-Pirouley, Thorsten Jänisch, Sarah Böringer, Patrick Lacroix-Desmazes

**Affiliations:** 1 ICGM, University Montpellier, CNRS, ENSCM, 34293 Montpellier, France; andrea1.ruiu@gmail.com (A.R.); jenli87@gmail.com (W.S.J.L.); 2 INCDO-INOE 2000, Research Institute for Analytical Instrumentation, ICIA, 400293 Cluj-Napoca, Romania; marin.senila@icia.ro; 3 IPREM, Université de Pau et des Pays de l’Adour, E2S-UPPA, CNRS, 64053 Pau, France; dominique.foix@univ-pau.fr; 4 Heraeus Deutschland GmbH & Co. KG, Heraeusstr. 12-14, 63450 Hanau, Germany; bernhard.bauer-siebenlist@heraeus.com; 5 Innovation Fluides Supercritiques, Batiment INEED, 26300 Alixan, France; k.seaudeau@supercriticalfluid.org; 6 Fraunhofer Institute for Chemical Technology, 76327 Pfinztal, Germany; tjaenisch82@gmail.com (T.J.); sarah.boeringer@ict.fraunhofer.de (S.B.)

**Keywords:** catalyst extraction, supercritical CO_2_, palladium recycling, fluoropolymers, complexing polymers, sustainable chemistry, circular economy

## Abstract

Precious metals such as palladium (Pd) have many applications, ranging from automotive catalysts to fine chemistry. Platinum group metals are, thus, in massive demand for industrial applications, even though they are relatively rare and belong to the list of critical materials for many countries. The result is an explosion of their price. The recovery of Pd from spent catalysts and, more generally, the development of a circular economy process around Pd, becomes essential for both economic and environmental reasons. To this aim, we propose a sustainable process based on the use of supercritical CO_2_ (i.e., a green solvent) operated in mild conditions of pressure and temperature (*p* = 25 MPa, T = 313 K). Note that the range of CO_2_ pressures commonly used for extraction is going from 15 to 100 MPa, while temperatures typically vary from 308 to 423 K. A pressure of 25 MPa and a temperature of 313 K can, therefore, be viewed as mild conditions. CO_2_-soluble copolymers bearing complexing groups, such as pyridine, triphenylphosphine, or acetylacetate, were added to the supercritical fluid to extract the Pd from the catalyst. Two supported catalysts were tested: a pristine aluminosilicate-supported catalyst (Cat D) and a spent alumina supported-catalyst (Cat A). An extraction conversion of up to more than 70% was achieved in the presence of the pyridine-containing copolymer. The recovery of the Pd from the polymer was possible after extraction, and the technological and economical assessment of the process was considered.

## 1. Introduction

Precious metals have played key roles in industry for many years, with the platinum group metals being seen as indispensable due to their unique physical and chemical properties [1,2]. Platinum group metals (including platinum, palladium, rhodium, iridium, and ruthenium) are rare and have high economic value. They find applications in the automotive, chemical, petroleum, electrical, and electronic industries. The raw ore of the platinum group metals are mainly located in South Africa and Russia. However, their production fell by 11% in South Africa as a result of lockdowns linked to the COVID-19 pandemic combined with an increase in labor and electricity costs [3]. At the same time, a growing demand of precious metals is observed every year due to industrial applications. It is worth noting that the demand for palladium (Pd) has increased from 242 tons in 2010 to over 305 tons in 2023, with the Pd supply and demand balance in the negative [4,5]. In addition, the total production of Pd from mines worldwide decreased in 2020 compared to 2019 [6]. In 2018, the price of Pd surpassed that of platinum and even exceeded that of gold in 2019 [7]. In this context, it becomes, therefore, essential to develop a circular economy process around Pd, including its recovery from spent catalysts. Metals can be reused an infinite number of times with their chemical and physical native properties unchanged. Metals available at the end of the recycling process are, therefore, equivalent to those obtained from mining. The recovery of catalysts usually requires multistep treatment processes by mechanical, chemical, and thermal unit operations [8]. Various techniques are already used at the industrial scale to recover precious metals from spent catalysts [9,10], which mainly consist of pyrometallurgy, hydrometallurgy, and leaching methods [11,12,13,14]. However, these processes require very high temperatures (>1273 K) or generate large amounts of leaching solvents. The development of lower energy-consuming, cheaper, and environmentally friendly methods for the recovery and recycling of precious metals is, therefore, challenging. Recently, attention has been focused on other methods, including processes using ionic liquids [15,16], polyphosphonate [17], ion exchange resins [18], or solvent extraction [19,20]. They exhibit good extraction capacities, but acidic solutions or waste pre-treatments are often required to achieve the objective.

A greener alternative consists of using supercritical CO_2_ (scCO_2_) as the extraction medium. CO_2_ is easily and highly available, possesses a tunable solvent power, and its supercritical domain can be achieved at mild conditions (T_c_ = 304 K, P_c_ = 7.38 MPa). These characteristics make scCO_2_ a green and inexpensive solvent for industrial applications. However, as a non-polar solvent, scCO_2_ alone is incapable of solubilizing metals and performing metal extraction. The presence of additives is, therefore, necessary. Recently, our group has proposed the use of scCO_2_-soluble metal-complexing copolymers to extract metals from solid matrices [21,22,23]. Even though some polymers such as siloxane [24,25] or vinylalkanoate-based [26,27] polymers are soluble in scCO_2_, they usually present some limitations. For instance, their solubility in scCO_2_ can be limited by their molecular weight and the nature of the chain-ends [28,29]. To avoid these drawbacks, we have developed systems using fluorinated monomers, namely 1,1,2,2-tetrahydroperfluorodecylacrylate (FDA), as a CO_2_-philic fragment [30,31]. These were associated with various complexing moieties (pyridine, triphenylphosphine, and acetylacetate). To ensure a better solubility of the copolymers in scCO_2_ and enable the use of lower CO_2_ pressures, gradient copolymers were preferred to block copolymers [32]. The ability of two copolymers, poly(4-vinyl pyridine-*co*-1,1,2,2-tetrahydroperfluorodecylacrylate) (P(4VP-*co*-FDA)) and poly(diphenylphosphinestyrene-*co*-1,1,2,2-tetrahydroperfluorodecylacrylate) (P(DPPS-*co*-FDA)), to extract up to 50% of Pd from alumina-supported catalysts was demonstrated [21,22,23,33]. The use of molecular complexing additives, such as dithiocarbamates, beta-diketones, dithizone, and perfluorocarboxylic acids, is also possible and has been reported in the literature [34,35,36,37,38,39]. However, molecular complexing agents appear to be less efficient than complexing copolymers to extract metals, although they allow avoiding the synthesis step of polymers. For instance, some molecular complexing agents such as fluorinated AOT (sodium bis (2,2,3,3,4,4,5,5-octafluoro-1-pentyl)-2-sulfosuccinate) [40], Cyanex 302/NaDDC (sodium diethyldithiocarbamate) [41], or Cyanex 272/NaDDC [41] were used for the extraction of metals. High (additive/metal) wt. ratios, from 20,700 for (Cyanex 302/NaDDC) to 34,500 in the case of F-AOT, were required to reach extraction yields of *ca* 92%. Complexing unit/metal molar ratios were also high, from 3137 to 6400, in the case of F-AOT and (Cyanex 272/NaDDC), respectively. Our group showed that the use of fluorinated copolymers bearing complexing units enabled a drastic reduction in the (additive/metal) wt. ratio as well as the complexing unit/metal ratio [42,43]. Thus, extraction yields from 50 to 96% were achieved with (additive/metal) wt. ratios of 4000 and 7200, and complexing unit/metal ratios equal to 99 or 830, respectively. In addition, fluorinated-based copolymers are highly soluble in scCO_2_.

In the present study, we show the ability of the fluorinated homopolymer PFDA and copolymers poly(acetoacetoxyethyl methacrylate-*co*-1,1,2,2-tetrahydroperfluorodecylacrylate) (P(AAEM-*co*-FDA)) and P(DPPS-*co*-FDA) to extract Pd from a pristine aluminosilicate-supported catalyst, Cat D, sometimes after a pre-treatment of reduction (Cat D-red) or oxidation (Cat D-red-ox). This scCO_2_ extraction process represents a safe and green method, while avoiding the use of high temperatures and the generation of polluting effluents. For the sake of comparison, we also included in this manuscript the previously published extraction results of Pd supported on catalysts Cat D, Cat D-red, and Cat D-red-ox in the presence of copolymer P(4VP-*co*-FDA) [22]. In addition, we showed the ability of the fluorinated copolymers P(DPPS-*co*-FDA) and P(4VP-*co*-FDA) to extract Pd from a spent alumina-supported catalyst, Cat A, also sometimes after a pre-treatment of oxidation (Cat A-red-ox). The recovery of the Pd metal from the polymer after extraction was also studied. Lastly, technological and economical assessments of the process are presented.

## 2. Results and Discussion

### 2.1. Presentation of the Catalysts Cat D and Cat A

#### 2.1.1. Catalyst Cat D

The pristine aluminosilicate-supported catalysts Cat D, Cat D-red, and Cat D-red-ox were precisely described by our group in a previous work [22]. The Cat D catalyst contains 2 wt% Pd in the form of PdO (100%) and its total carbon value is equal to 0%. Its pre-treated version, Cat D-red, was obtained by reduction under H_2_ to give a catalyst composition of Pd^0^ (79%) with a minor amount of PdO (21%). Reduction by H_2_ followed by oxidation with Cl_2_ leads to catalyst Cat D-red-ox with a composition of Na_2_PdCl_4_ (85%) and PdO (15%) (Figure S5).

#### 2.1.2. Catalyst Cat A

**Figure 1:**
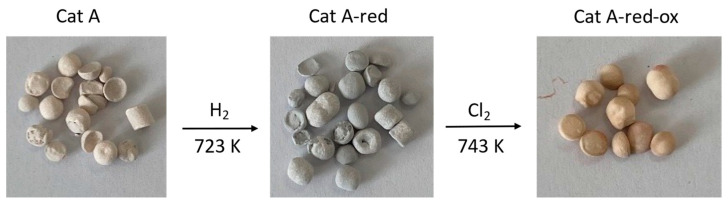
Images of the Pd-supported catalysts Cat A, Cat A-red, and Cat A-red-ox.

Different analytical techniques were used to characterize the spent catalysts Cat A, Cat A-red, and Cat A-red-ox. ICP-OES and XPS were used to determine the concentration of the supported Pd and its oxidation state as well as the nature of the Pd species, respectively. The catalyst porosity was estimated by BET. The spatial distribution of the metal in the catalyst and the metal particle size were analyzed by SEM-EDX and TEM, respectively. All the catalysts are supported on an α-alumina carrier and contain 0.5 wt% of Pd (Table S10). The average pore size of the alumina support is estimated at 105–335 nm. The three catalysts Cat A differ in the Pd oxidation states and the nature of the Pd species on the support (XPS). In order to evaluate the efficiency of the extraction process on different Pd species, the oxidation state and the nature of the Pd species in the different catalysts were modified by a pre-treatment (Figure 1). Note that the catalyst support (α-alumina) and metal distribution were kept unchanged.

Thus, Cat A mainly contains palladium oxide PdO (100%) (Figure S17). The average particle size of the palladium nanoparticles is 2.0 nm (Figure S23). Its total carbon content is 1.3%. Pre-treatment with H_2_ leads to Cat A-red containing Pd^0^ (71%) and PdO (29%) (Figure S18). Finally, further chlorination gives Cat A-red-ox containing mainly Pd-chloride, presumably PdCl_2_ (89%) with a minor amount of PdO (11%) (Figure S19). An increase of the average nanoparticle size of up to 5.9 nm was observed for Cat A-red-ox (Figure S24).

### 2.2. Pd Extraction from Catalysts Cat D and Cat A with scCO_2_-Soluble (co)Polymers

#### 2.2.1. Synthesis of the Fluorinated scCO_2_-Soluble (co)Polymers Capable of Complexing with Pd

The CO_2_-soluble (co)polymers composed of fluorinated CO_2_-philic units associated with complexing units (able to interact with metals such as Pd) were synthesized.

**Figure 2:**
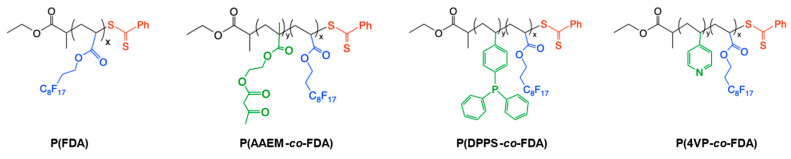
Structures of the CO_2_-soluble copolymers bearing metal-complexing groups.

A polymer poly(1,1,2,2-tetrahydroperfluorodecylacrylate) (P(FDA)) with a targeted molecular weight of 5000 g/mol and three gradient copolymers with targeted molecular weights of 10,000 g/mol were synthesized by reversible addition-fragmentation chain-transfer (RAFT) polymerization [44]. The gradient copolymers were composed of different monomers bearing complexing groups (acetoacetoxyethyl methacrylate (AAEM), 4-(diphenylphosphino)styrene (DPPS), 4-vinyl pyridine (4VP)), and fluorinated monomer units (FDA) to ensure their good solubility in scCO_2_ (Figure 2).

The synthesis of gradient copolymers was possible due to the reactivity ratio of the different monomers. The Alfrey and Price Q and e values of FDA [45], 4VP [46], and AAEM [47] can be found in the literature. Regarding DPPS, the Q and e values of chloromethyl styrene (CMSty) have been considered as a first approximation [48]. The Alfrey and Price equations were then used to calculate the reactivity ratios from these Q and e values [46]: r_AAEM_ = 1.61 and r_FDA_ = 0.56, r_CMSty_ = 1.41 and r_FDA_ = 0.24, r_4VP_ = 4.05 and r_FDA_ = 0.21. Thus, the polymer chains were first enriched with complexing units, implying the gradient structure of the copolymers P(AAEM-*co*-FDA), P(DPPS-*co*-FDA), and P(4VP-*co*-FDA). The syntheses and characterization of these (co)polymers have been described by our group in a previous work (Table S15) [33]. More precisely, the homopolymer PFDA (M_n_ = 5850 g/mol) exhibiting 11 FDA monomer units was first synthesized and is soluble in scCO_2_. The other complexing copolymers P(AAEM-*co*-FDA) (M_n_ = 13,500 g/mol), P(DPPS-*co*-FDA) (M_n_ = 11,600 g/mol), and P(4VP-*co*-FDA) (M_n_ = 11,800 g/mol) contain an average of 19 acetoacetoxy units, 7 triphenylphosphine units, and 20 pyridine units, respectively. This allows for good complexing ability, while ensuring the solubility of the copolymers in scCO_2_ (provided by 18 FDA monomer units) under mild conditions of temperature and pressure (see SI Section S3). It is noteworthy that a keto–enol equilibrium exists in the case of the copolymer P(AAEM-*co*-FDA). The acetoacetoxy complexing group can be activated in the presence of a slight excess of 1,1,3,3-tetramethylguanidine (TMG) to form the enolate group, which is a better ligand for metals (Figure S27) [49].

In order to verify the solubility of the (co)polymers under the experimental conditions, their cloud point measurements were performed in dense CO_2_ (Figure S30). All the (co)polymers were found to be soluble under mild conditions (i.e., *p* < 27 MPa in the temperature range of 298 to 338 K), which represents a major advantage for the application of the copolymers in metal extraction [22,32,33,50]. The cloud point curves enabled establishing the following increasing order of solubility of the (co)polymers: P(DPPS-*co*-FDA) ≅ P(4VP-*co*-FDA) < P(AAEM-*co*-FDA) < P(FDA).

#### 2.2.2. Pd Extraction from Catalysts Cat D with scCO_2_-Soluble (co)Polymers

The extraction procedure was performed under mild supercritical operating conditions (*p* = 25 MPa and T = 313 K), which renders the metal extraction process compatible with commercial applications. Indeed, the decaffeination of coffee by scCO_2_ is routinely operated under the same range of conditions at the industrial scale (i.e., at a pressure of 22 MPa and T = 363 K) [51]. In addition, performing the extraction process at 25 MPa and 313 K enabled operating above the cloud point curve of the (co)polymers and, therefore, ensured their good solubilization in scCO_2_ during the process.

The inability of scCO_2_ alone to extract Pd from Cat D catalysts (before and after treatment) was first verified as blank experiments. With less than 3% extraction, it clearly appears that, without a polymer, scCO_2_ was unable to remove Pd from the catalyst supports due to the negligible solubility of Pd species in neat scCO_2_ and to the quasi absence of chemical interaction between metal and nonpolar CO_2_ (Table 1 Run E1–E3; Tables S2 and S3 Run E1–E3 and Figure S2). The presence of (co)polymers as a complexing agent to facilitate the solubilization of Pd in scCO_2_ is, therefore, required for such metal extraction.

**Figure 3:**
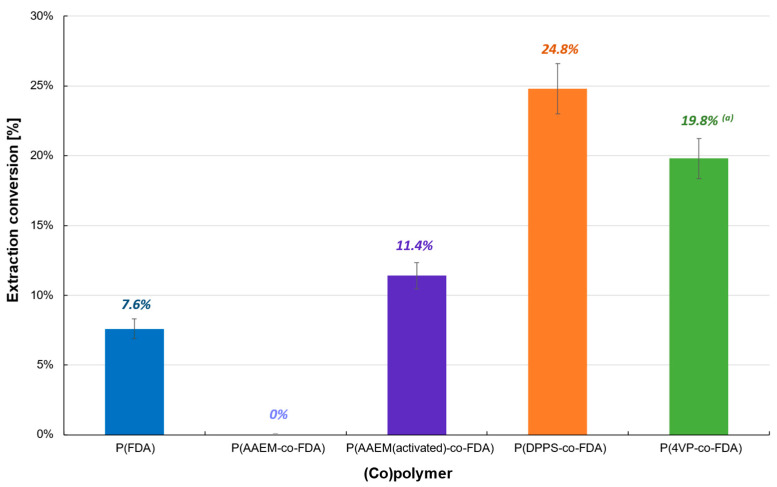
Extraction of palladium from Cat D in the presence of complexing (co)polymers P(FDA_11_), P(AAEM_19_-*co*-FDA_18_), P(AAEM_(activated)19_-*co*-FDA_18_), P(DPPS_7_-*co*-FDA_18_), and P(4VP_20_-*co*-FDA_18_); ^(a)^ result already presented in a previous work [22].

The first polymer-based extraction experiments were carried out at 313 K and 25 MPa on Cat D in the presence of the four synthesized (co)polymers (Table 1). The oxide form of PdO renders the extraction on Cat D more difficult, which resulted in a low extraction conversion (<25%) (Table 1, Figure 3, Tables S2 and S3). P(AAEM-*co*-FDA) was unable to extract PdO, but a slight improvement was observed after activation of the acetoacetoxy complexing group, resulting in 11.4% of Pd extracted. However, similar results were obtained with the homopolymer P(FDA). The most promising extraction results were observed in the presence of copolymers P(4VP-*co*-FDA) and P(DPPS-*co*-FDA), with Pd extractions of 19.8 and 24.8%, respectively. Even though the level of extraction remains low, it demonstrates that the organic ligands pyridine and triphenylphosphine have better reactivity towards such Pd derivatives.

**Figure 4:**
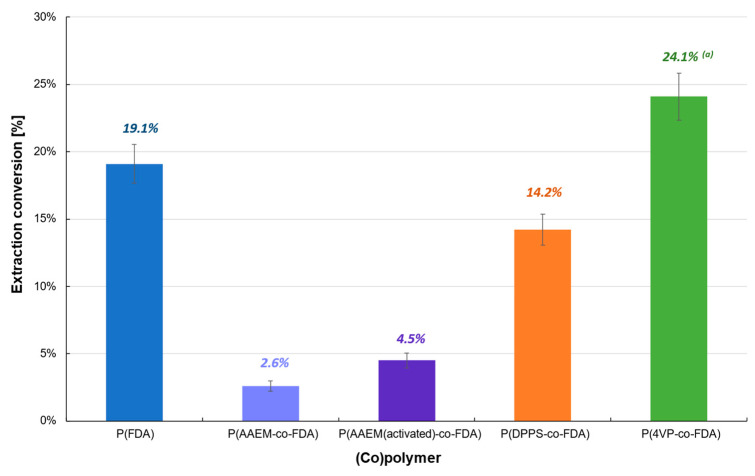
Extraction of palladium from Cat D-red in the presence of complexing (co)polymers P(FDA_11_), P(AAEM_19_-*co*-FDA_18_), P(AAEM_(activated)19_-*co*-FDA_18_), P(DPPS_7_-*co*-FDA_18_), and P(4VP_20_-*co*-FDA_18_); ^(a)^ result already presented in a previous work [22].

Extractions performed on the reduced catalyst (Cat D-red), containing mainly Pd^0^, also showed poor efficiency (<25%) (Table 1, Figure 4, Tables S2 and S3). The best extraction conversion (24.1%) was obtained using P(4VP-*co*-FDA), whereas an extraction of only 14.2% was obtained with P(DPPS-*co*-FDA), contrary to Cat D. Therefore, the use of copolymer P(4VP-*co*-FDA) enabled the improvement of the extraction by almost 70%. Homopolymer P(FDA) was also able to extract Pd^0^ (19.1%). In contrast, both forms of copolymer P(AAEM-*co*-FDA) (non-activated and activated) were incapable of extracting Pd^0^. The low efficiency of the (co)polymers to interact with the metal and remove it from the supported catalyst can be explained by the low or even non-existent affinity of the metal complexing units with respect to the Pd^0^ nanoparticles.

**Figure 5:**
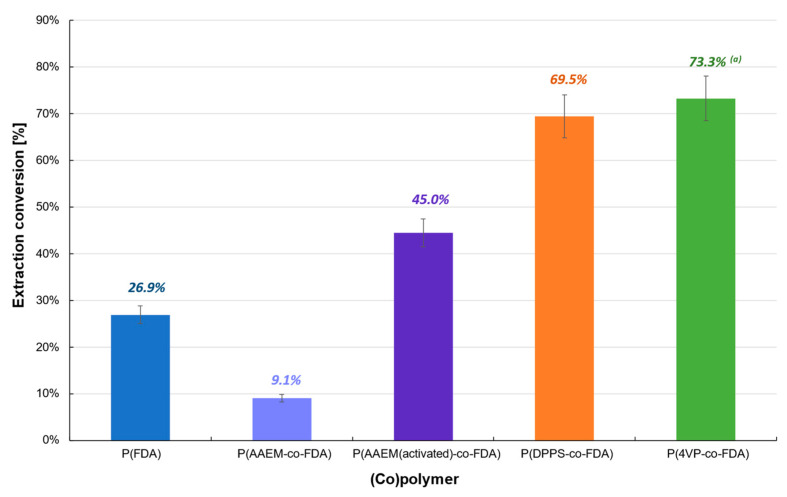
Extraction of palladium from Cat D-red-ox in the presence of complexing (co)polymers P(FDA_11_), P(AAEM_19_-*co*-FDA_18_), P(AAEM_(activated)19_-*co*-FDA_18_), P(DPPS_7_-*co*-FDA_18_), and P(4VP_20_-*co*-FDA_18_); ^(a)^ result already presented in a previous work [22].

Finally, the (co)polymer extraction procedure was tested with the catalyst Cat D-red-ox containing mainly Na_2_PdCl_4_ (Table 1, Figure 5, Tables S2 and S3). The extraction conversion remains low with the homopolymer P(FDA) (26.9%) or almost non-existent with P(AAEM-*co*-FDA) copolymer (9.1%). However, after activation of the latter with TMG, the extraction conversion of Cat D-red-ox exhibits an increase of 394%. It is worth noting that the extraction experiments in the presence of P(DPPS-*co*-FDA) and P(4VP-*co*-FDA) copolymers led to a significant efficiency, enabling the removal of 69.5 and 73.3% of the Pd absorbed on the catalyst, respectively.

**Figure 6:**
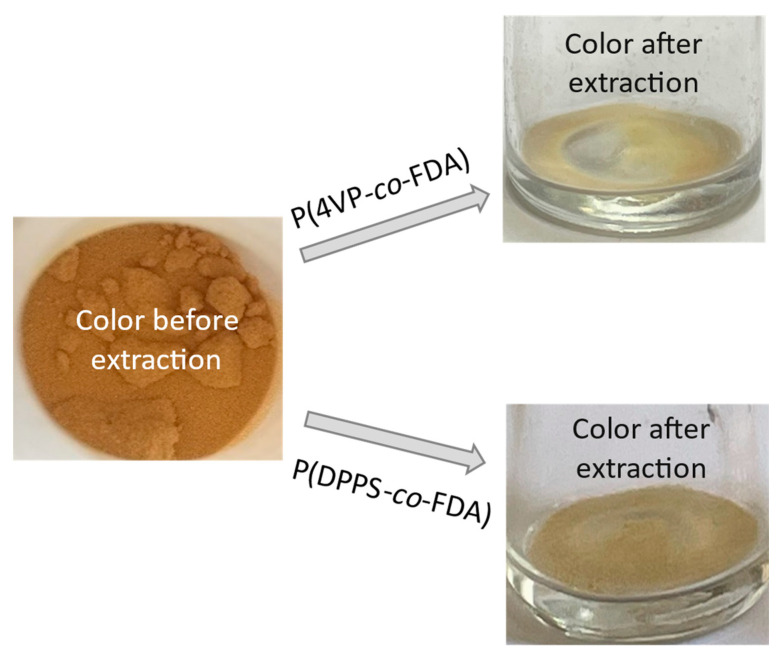
Change of color of the supported catalyst Cat D-red-ox after scCO_2_ extraction of Pd assisted by the copolymers P(4VP-*co*-FDA) (top right) and P(DPPS-*co*-FDA) (bottom right) at 313 K and 25 MPa.

A colour change of the supported Cat D-red-ox catalyst, from reddish-brown to yellowish, was observed after extraction treatment (Figure 6). This suggests that the colored Pd species were removed from the catalyst support and confirms the success of the Pd extraction.

It seems that Na_2_PdCl_4_ halogenated species have a higher reactivity with phosphine or pyridine ligands than the oxide (PdO) or metallic (Pd^0^) species found on Cat D or Cat D-red catalysts. Halogenated Pd derivatives are usually used as precursors in organometallic chemistry to synthesize novel Pd-based complexes [52,53,54,55,56]. The formation and solubility, in scCO_2_, of a complex between a fluorinated complexing copolymer and Pd(II) salt was demonstrated, in a previous work, with the help of a high pressure UV cell [49]. In situ UV analyses were performed and exhibited the presence of the band at 378 nm, which confirms the formation of a Pd(II)-complex. Furthermore, both P(DPPS-*co*-FDA) and P(4VP-*co*-FDA) showed the best extraction results with all the catalysts, even though the highest efficiency was obtained with Cat D-red-ox. Copolymers P(DPPS-*co*-FDA) and P(4VP-*co*-FDA) appear to be good candidates for solubilizing the Pd/polymers species and transporting them in the scCO_2_ medium. As demonstrated, phosphine and pyridine ligands ensure a good complexation between the Pd species and the CO_2_-soluble copolymers. Such encouraging results make this polymer-assisted extraction method of Pd from the aluminosilica support of the catalyst very promising.

#### 2.2.3. Pd Extraction from Catalysts Cat A with scCO_2_-Soluble (co)Polymers

**Figure 7:**
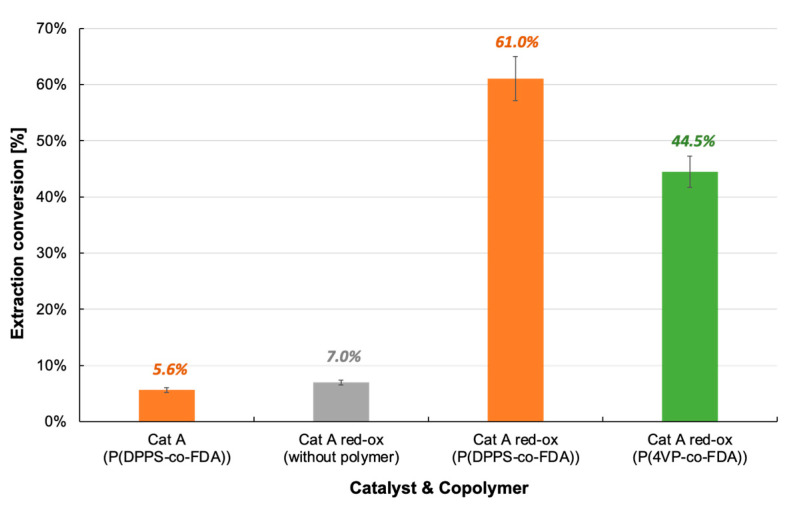
Extraction of palladium from Cat A and Cat A-red-ox with and without complexing copolymer P(DPPS-*co*-FDA) and P(4VP-*co*-FDA).

The same scCO_2_ extraction procedure used for pristine Cat D was applied to spent catalyst Cat A (i.e., *p* = 25 MPa and T = 313 K). With copolymers P(DPPS-*co*-FDA) and P(4VP-*co*-FDA) giving better extraction results on Cat D catalysts, we focused on these two copolymers for this study. As with previous observations, the nature of the catalyst has a strong influence on the extraction efficiency. Surprisingly, poor extraction results were obtained in the presence of P(DPPS-*co*-FDA). Only 6% of Pd was removed on Cat A, whereas almost 25% extraction was observed on Cat D with the same copolymer, even though both catalysts contained PdO species (Table 1 vs. Table 2, Figure 3 vs. Figure 7). The main difference between the two catalysts is their carbon content. In addition to the poor reactivity of such Pd derivatives towards the phosphine ligands, the fact that the spent catalyst Cat A contains 1.3% of carbon contrary to the pristine catalyst Cat D may explain this result.

The extraction procedure with and without P(DPPS-*co*-FDA) was tested in the presence of chlorinated catalyst Cat A-red-ox (Table 2, Figure 7, Tables S5–S7). With only 7% Pd extracted from the catalyst in the absence of the copolymer, the key role of the copolymer in the extraction process was confirmed. The extraction efficiency increased by 771% when the experiment was performed in the presence of the copolymer P(DPPS-*co*-FDA). With the removal of 61% of the Pd absorbed on the catalyst, copolymer P(DPPS-*co*-FDA) proves its ability to form complexes with halogenated Pd. P(4VP-*co*-FDA) also proved capable of extracting PdCl_2_ from the support, although Pd extraction was lower at 44.5%.

### 2.3. Recovery of the Pd from the Polymer/Pd Complex

**Figure 8:**
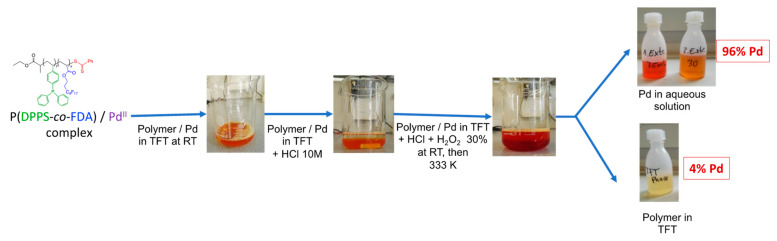
General recovery process of the Pd from the polymer/Pd complex.

The recovery of the Pd metal is important from both an economical and environmental point of view due to the overconsumption of metals over the last decades. With the need of green and sustainable development, the catalyst and, in our case, the polymer, must have a long product lifetime and be easily recyclable to be considered as a viable option in industry [57,58]. A first study was performed to verify the possibility of isolating the Pd from the copolymer after the extraction process. As already mentioned (vide supra), the copolymer-Pd(II) complex was soluble in scCO_2_. It was, thus, recovered at the outlet of the extraction set-up, during the rinsing step, by bubbling into a flask containing deionized water (see SI Section 1 and Figure S1). In order to separate the Pd from the copolymer, the P(DPPS-*co*-FDA)/Pd(II) complex was dissolved in trifluoro toluene (TFT) at room temperature. Hydrochloric acid and hydrogen peroxide were then added, generating a water soluble [PdCl_6_]^2−^ complex. The typical orange-red color of the [PdCl_6_]^2−^ complex [59] was found to intensify in the aqueous solution. The mixture was heated to 353 K to decompose excess hydrogen peroxide and the aqueous Pd solution was separated from the colorless TFT solution. A second extraction step of the organic phase at 333 K with hydrochloric acid resulted in a total Pd recovery of 96% according to ICP-OES measurements (Figure 8). The residual 4% Pd was analyzed by ICP-OES in the TFT copolymer solution. Chloropalladate solutions are standard intermediates in palladium refining and can be further processed to Pd metal by a known method [60]. Furthermore, the isolated copolymer can be used for a new extraction cycle after the evaporation of TFT.

These first results proved successful as they showed the possibility of isolating the Pd from the copolymer. The proposed method for the recovery of palladium and the copolymer is a very promising technique. Further studies are planned to verify the quality of the recovered Pd and evaluate the performance of the recycled copolymers.

### 2.4. Technological and Economical Assessment

Although the presented Pd recovery yields are too low for an industrial realization, a technological and economical assessment in comparison to an industrial leaching process was estimated. The purpose was to identify the main factors that influence the overall economical impact. For this purpose, the pre-treatment of the catalysts, the supercritical CO_2_ extraction, and post-treatment (recovery of Pd after the CO_2_ extraction process) were included for the evaluation. Laboratory data for these processes were used and compared to an industrial process.

The extractive process of this work considers 1250 kg of catalyst treated per day at 25 MPa and 313 K with a CO_2_ to feed ratio of 15 (kg of CO_2_ for kg of raw material treated). The residence time inside the autoclave was fixed at 3 h. The size of the autoclave considered is 2 × 500 L with CO_2_ recirculation. Under these conditions, the required set-up leads to a cost per kg of catalyst treated of about 1–3 €/kg. For the supercritical CO_2_ extraction process, CAPEX (capital expenditure) per kg is in the range of a conventional leaching process. Therefore, from a technological view and including the above premise on recovery yield, this new process appears to be feasible. As anticipated, the operative expenses are much higher than the existing industrial process. A closer look into the operating costs (OPEX, operational expenditure) reveals the main factors for an economical success. Once the Pd recovery yield has been improved, the polymer extractant production and recycling is of fundamental importance. The majority of the cost of chemicals (86%) is attributed to the synthesis of the complexing copolymer, taking into account a 99% polymer recycling rate. The polymer-extracting agent is a non-commercial product and, so, the cost has been estimated based on a laboratory-scale production. A production on an industrial scale would help to decrease this economic factor.

Electrical power is the second-largest influence on the overall costs. Mainly, the pre-treatment process at high temperatures and the recycling of the solvent from the post-treatment will be the first targets for an optimization.

The process time, which plays an important role in Pd recycling, is comparable to the current industrial process and is acceptable for an industrial application.

A life-cycle assessment (LCA) was also conducted to compare both processes. The necessary data were gathered from the laboratory-scale extraction process, as well as the existing industrial process. The difference in the maturity of both processes leads to results that are, by far, in favor of the existing industrial process. The comparison provided similar options for the future development of the extraction process. At first place, this is the efficient production and recycling of the polymer extractant. An increase in the scale of the recovery processes should lead to a more resource-efficient post-treatment of the polymer–Pd complex.

## 3. Materials and Methods

The pristine catalyst (Cat D) and the pre-treated catalysts Cat D-red and Cat D-red-ox, as well as the spent catalyst (Cat A) and the pre-treated catalysts Cat A-red and Cat A-red-ox were provided by Heraeus Deutschland GmbH & Co (Hanau, Germany). Cat D-red was obtained from catalyst D after reduction over 4 h under H_2_ atmosphere at 773 K. Catalyst D gave Cat D-red-ox after reduction, followed by oxidation over 4 h under Cl_2_ atmosphere at 743 K (Figure S5). Cat A-red was obtained from catalyst Cat A after reduction over 5 h under H_2_ atmosphere at 723 K. Catalyst A gave Cat A-red-ox after reduction, followed by oxidation over 5 h under Cl_2_ at 743 K (Figure 1). Inductively coupled plasma optical emission spectrometry (ICP-OES), X-ray photoelectron spectroscopy (XPS), transmission electron microscopy (TEM), scanning electron microscopy with energy dispersive X-ray spectroscopy (SEM-EDX), and nitrogen adsorption–desorption isotherms (BET) were used to characterize the catalysts. The results of these different techniques were reported in the Supporting Information (SI). Carbon dioxide (CO_2_, SFE 5.2, Air Liquide, 99.9%) was used as received. The polymer syntheses were performed according to the procedures described in the SI, in accordance with a previous work [33]. The polymer characterization was also shown in the SI. The procedures for the extraction of Pd with catalysts Cat D, Cat D-red, and Cat D-red-ox (E1-E18) and Cat A-red-ox (E22), performed by ICGM, and catalysts Cat A and Cat A-red-ox (E19-E21), performed by ICT, are detailed in the SI, accompanied by the calculations of conversion and uncertainty.

## 4. Conclusions

A promising way for the extraction of Pd from pristine and spent supported catalyst in scCO_2_ under mild conditions (*p* = 25 MPa and T = 313 K) with various CO_2_-philic complexing (co)polymers was presented. Whichever the catalyst used (pristine or spent), the best extraction results were obtained in the presence of the oxidized forms of the catalysts (Cat D-red-ox and Cat A-red-ox), mainly composed of Pd(II) halogenated species. The nature of the copolymer also plays an essential role in the extraction efficiency. Thus, copolymers containing pyridine and triphenylphosphine units (P(4VP-*co*-FDA) and P(DPPS-*co*-FDA), respectively) gave extraction conversions between *ca* 60 and more than 70%. Recovery of the Pd and copolymers was also possible after extraction. However, the technological and economical assessment showed that this process was not, in its current state, capable of competing with a conventional leaching process. The polymer production is remaining a penalty point for the OPEX of the whole extraction process. It is worth noting that this work is conducted at a laboratory scale and further studies will be pursued to improve the extraction process. Nevertheless, the results presented in this study show a promising alternative to Pd precious metal recovery by means of an environmentally friendly and non-destructive way, avoiding high-energy demanding procedures or the generation of hazardous effluents.

## Figures and Tables

**Table 1:** Polymer-assisted extractions of Pd from aluminosilica-supported catalysts (Cat D) in scCO_2_ at T = 313 K and *p* = 25 MPa ^(a)^.

Run	Catalyst	(Co)polymer	Complexing Group	Additive	Polymer/Pd Molar Ratio	Complexing Group/Pd Molar Ratio	Additive/Pd Molar Ratio	Additive/ Complexing Group Ratio	Extracted Pd from the Support of the Catalyst [%] ^(b)^
**E1 ^(e)^**	Cat D	None	-	-	-	-	-	-	0.6
**E2 ^(e)^**	Cat D-red	None	-	-	-	-	-	-	3.2
**E3 ^(e)^**	Cat D-red-ox	None	-	-	-	-	-	-	1.5
**E4**	Cat D	P(FDA_11_) ^(c)^	RAFT end group	-	5.735	5.735	-	-	7.6
**E5**	Cat D-red	P(FDA_11_) ^(c)^	RAFT end group	-	10.116	10.116	-	-	19.1
**E6**	Cat D-red-ox	P(FDA_11_) ^(c)^	RAFT end group	-	4.482	4.482	-	-	26.9
**E7**	Cat D	P(AAEM_19_-*co*-FDA_18_) ^(a)^	AAEM	-	0.445	8.450	-	-	0.0
**E8**	Cat D	P(AAEM_19_-*co*-FDA_18_) ^(a)^	AAEM_activated_	TMG	0.461	8.754	10.002	1.143	11.4
**E9**	Cat D-red	P(AAEM_19_-*co*-FDA_18_) ^(a)^	AAEM	-	0.481	9.134	-	-	2.6
**E10**	Cat D-red	P(AAEM_19_-*co*-FDA_18_) ^(a)^	AAEM_activated_	TMG	0.481	9.142	10.446	1.143	4.5
**E11**	Cat D-red-ox	P(AAEM_19_-*co*-FDA_18_) ^(a)^	AAEM	-	0.465	8.831	-	-	9.1
**E12**	Cat D-red-ox	P(AAEM_19_-*co*-FDA_18_) ^(a)^	AAEM_activated_	TMG	0.449	8.532	10.244	1.201	44.5
**E13**	Cat D	P(DPPS_7_-*co*-FDA_18_) ^(d)^	DPPS	-	2.086	14.600	-	-	24.8
**E14**	Cat D-red	P(DPPS_7_-*co*-FDA_18_) ^(d)^	DPPS	-	2.065	14.457	-	-	14.2
**E15**	Cat D-red-ox	P(DPPS_7_-*co*-FDA_18_) ^(d)^	DPPS	-	2.093	14.654	-	-	69.5
**E16 ^(e)^**	Cat D	P(4VP_20_-*co*-FDA_18_) ^(a)^	4VP	-	0.557	11.148	-	-	19.8
**E17 ^(e)^**	Cat D-red	P(4VP_20_-*co*-FDA_18_) ^(a)^	4VP	-	0.553	11.068	-	-	24.1
**E18 ^(e)^**	Cat D-red-ox	P(4VP_20_-*co*-FDA_18_) ^(a)^	4VP	-	0.560	11.201	-	-	73.3

^(a)^ General conditions: m_catalyst_ ≈ 200 mg, m_polymer_ ≈ 250 mg (see Table S2), m_CO2, batch step_ = 35 g, m_CO2, flushing_ step = 145 g; ^(b)^ determined by inductively coupled plasma—optical emission spectrometry (ICP-OES); ^(c)^ m_polymer_ = 1.28 g (E4), m_polymer_ = 2.28 g (E5), m_polymer_ = 1.01 g (E6); ^(d)^ m_polymer_ = 0.92 g (E13), m_polymer_ = 0.93 g (E14), m_polymer_ = 0.93 g (E15); ^(e)^ result already presented in a previous work [22].

**Table 2:** Polymer-assisted extractions of Pd from α-alumina-supported catalysts (Cat A) in scCO_2_ at T = 313 K and *p* = 25 MPa ^(a)^.

Run	Catalyst	(Co)polymer	Complexing Group	Additive	Polymer/Pd Molar Ratio	Complexing Group/Pd Molar Ratio	Additive/Pd Molar Ratio	Additive/Complexing Group Ratio	Extracted Pd from the Support of the Catalyst [%] ^(b)^
**E19 ^(c)^**	Cat A	P(DPPS_7_-*co*-FDA_18_)	DPPS	-	1.68	11.75	-	-	5.6
**E20 ^(d)^**	Cat A-red-ox	-	-	-	-	-	-	-	7.0
**E21 ^(e)^**	Cat A-red-ox	P(DPPS_8_-*co*-FDA_24_)	DPPS	-	1.33	48.22	-	-	61.0
**E22 ^(f)^**	Cat A-red-ox	P(4VP_20_-*co*-FDA_18_)	4VP	-	0.48	9.65	-	-	44.5

^(a)^ General conditions: m_CO2, batch step_ = 215 g, m_CO2, flushing step_ = 600 g (see Table S5); ^(b)^ determined by inductively coupled plasma—optical emission spectrometry (ICP-OES); ^(c)^ m_catalyst_ = 575 mg, m_polymer_ = 525 mg, m_CO2, flushing step_ = 1500 g; ^(d)^ m_catalyst_ = 10.865 g, m_polymer_ = 0 g; ^(e)^ m_catalyst_ = 285 mg, m_polymer_ = 1.096 g; ^(f)^ m_catalyst_ = 510 mg, m_polymer_ = 135 mg.

## Data Availability

The data presented in this study are available in the Supporting Information.

## Supplementary Materials

The following supporting information can be downloaded at: https://www.mdpi.com/article/10.3390/molecules28176342/s1, Figure S1: Scheme of the ICGM extraction set-up; Figure S2: Extraction of palladium from Cat D in the absence of complexing polymer; Figure S3: Scheme of the ICT extraction apparatus; Figure S4: Picture of Cat D; Figure S5. Images of the Pd supported catalysts Cat D, Cat D-red, and Cat D-red-ox; Figure S6: Picture of Cat A; Figure S7: SEM-EDX image of Cat D; Figure S8: EDX and element percentage at the surface of Cat D; Figure S9: EDX and element percentage inside Cat D (fractured bead); Figure S10: Nitrogen adsorption–desorption isotherms and pore size distribution of Cat D; Figure S11: Nitrogen adsorption–desorption isotherms and pore size distribution of Cat D-red; Figure S12: Nitrogen adsorption–desorption isotherms and pore size distribution of Cat D-red-ox; Figure S13: XPS spectrum Pd 3d of Cat D; Figure S14: XPS spectrum Pd 3d of Cat D-red; Figure S15: XPS spectrum Pd 3d of Cat D-red-ox; Figure S16: XPS analysis comparison of Cat D-red-ox, PdCl_2_ and Na_2_PdCl_4_; Figure S17: XPS spectrum Pd 3d of Cat A; Figure S18: XPS spectrum Pd 3d of Cat A-red; Figure S19: XPS spectrum Pd 3d of Cat A-red-ox; Figure S20: TEM and particle size distribution of Cat D; Figure S21: TEM and particle size distribution of Cat D-red; Figure S22: TEM of Cat D-red-ox; Figure S23: TEM and particle size distribution of Cat A; Figure S24: TEM and particle size distribution of Cat A-red-ox; Figure S25: ^1^H-NMR spectrum (400 MHz, CFC-113 + C_6_D_6_ capillaries) of P(FDA) after precipitation; Figure S26: ^1^H-NMR spectrum (400 MHz, acetone-d_6_) of P(AAEM-*co*-FDA) after precipitation; Figure S27: Activation of acetoacetoxy complexing group in the presence of TMG to form the enolate group; Figure S28: ^1^H-NMR spectrum (400 MHz, CFC-113 + C_6_D_6_ capillaries) of P(DPPS-*co*-FDA) after precipitation; Figure S29: ^1^H-NMR spectrum (400 MHz, CDCl_3_) of P(4VP-*co*-FDA) after precipitation; Figure S30: Cloud point curves in dense CO_2_ of the (co)polymers used in this study at in dense (at a polymer concentration of ca. 1 wt% of polymer relative to CO_2_); Table S1: Sample nomenclature for the ICGM extraction; Table S2: Reactant ratios for extraction experiments performed with Cat D catalysts in supercritical CO_2_ at 313 K and 25 MPa; Table S3: Extraction results and errors of the reaction experiments performed with Cat D catalysts in supercritical CO_2_ at 313 K and 25 MPa; Table S4: Sample nomenclature for the ICT extraction; Table S5: Reactant ratios for extraction experiments performed with Cat A catalysts in supercritical CO_2_ at 313 K and 25 MPa; Table S6: Extraction results and errors of the reaction experiments performed with Cat A catalysts in supercritical CO_2_ at 313 K and 25 MPa; Table S7: Extraction results and errors of the reaction experiments performed with Cat A catalysts in supercritical CO_2_ at 313 K and 25 MPa; Table S8: Digestion program for ICP-OES samples; Table S9: Pd quantification in Cat D catalysts by ICP-OES; Table S10: Pd quantification in Cat A catalysts by ICP-OES; Table S11: Specific surface areas and average pore diameters determined by BET for Cat D; Table S12: Cat A structural characteristics; Table S13: Elemental composition of Cat D determined by XPS (atomic percentages); Table S14: Elemental composition of Cat A determined by XPS (atomic percentages); Table S15: Synthesis by RAFT polymerization of P(FDA) and gradients complexing copolymers [61,62,63].

## Sample Availability

Samples of the compounds are not available from the authors.
