# Supplementary material for: Recovery of Precious Metals: A Promising Process Using Supercritical Carbon Dioxide and CO2-Soluble Complexing Polymers for Palladium Extraction from Supported Catalysts
Source: Molecules. 2023 Aug 30;28(17):6342. doi: 10.3390/molecules28176342 (PMC10488959; doi:10.3390/molecules28176342)
Supplement: Supplementary file 1 [file molecules-28-06342-s001.zip › molecules-2509913-supplementary.pdf]

Recovery of precious metals: a promising process using supercritical carbon dioxide and CO_2-soluble complexing polymers for palladium extraction from supported catalysts

Andrea Ruiu,[1] W.S. Jennifer Li,[1] Marin Senila[2], Cécile Bouilhac,[1,*] Dominique Foix,[3] Bernhard Bauer-Siebenlist,[4] Karine Seaudeau-Pirouley,[5] Thorsten Jänisch,[6] Sarah Böringer[6] and Patrick Lacroix-Desmazes[1,*]

1. ICGM, Univ. Montpellier, CNRS, ENSCM, 34293 Montpellier, France;
2. INCDO INOE 2000, Research Institute for Analytical Instrumentation, ICIA, 400293 Cluj-Napoca, Romania;
3. IPREM, Université de Pau et des Pays de l'Adour, E2S-UPPA, CNRS, 64053 Pau, France;
4. Heraeus Deutschland GmbH & Co. KG, Heraeusstr. 12-14, 63450 Hanau, Germany
5. Innovation Fluides Supercritiques, Batiment INEED, 26300 Alixan, France;
6. Fraunhofer Institute for Chemical Technology, 76327 Pfinztal, Germany;

* Correspondence: patrick.lacroix-desmazes@enscm.fr;

cecile.bouilhac@umontpellier.fr

SUPPORTING INFORMATION

1. Extraction experimental procedure

1.1 Extraction general procedure for Cat D (and Cat A E22)

The catalyst and the polymer were placed in a 35 mL stainless steel extraction cell (Top Industrie, France), which was then tightly closed. The extraction cell was equipped with magnetic stirring, a PTFE-coated magnetic stir bar, a rupture disk, a pressure transducer, and two stainless steel filters (PORAL, class 7) disposed at the inlet and outlet of the set-up (Figure S1). The extraction cell was heated with a mantle monitored by a Proportional-Integral-Derivative temperature controller with a thermocouple (type K) inside the extraction medium. An ISCO model no. 260D automatic syringe pump (with an internal pressure transducer), thermostated by a water/isopropanol mixture delivered by a LAUDA RE206 circulating pump, was used to pressurize the extraction cell with carbon dioxide (CO_2, SFE 5.2, Air Liquide B50 bottle with plunger tube, 99.9%,). In the case of Cat D-red-ox, 0.02 mL of DIPEA (N,N-diisopropylethylamine) were added to catch any potential release of HCl during the extraction (chlorine in Na_2PdCl_4). The ISCO pump was stabilized at 27 MPa and 308 K (d_{CO2}=0.91308 g/mL). Afterwards, the extraction cell was filled with CO_2 until 25 MPa at 313 K were reached in the extraction cell (≈39 mL of CO_2 delivered by the ISCO pump, m_{CO2}≈35 g) (1 mL = 10^{-3} L and 1 g = 10^{-3} Kg in SI units). The extraction was performed under magnetic stirring at 100 rpm for 1 h (1 h = 3600 s in SI units) at 25 MPa and 313 K (batch conditions). The cell was then flushed with ≈160 mL of CO_2 delivered by the ISCO pump (26 MPa and 308 K in the ISCO pump, d_{CO2}=0.90729 g/mL, m_{CO2}≈145 g), at a flow rate of approximately 0.6-1.2 mL/min. The polymer was recovered at the outlet of the extraction set-up during the rinsing step by bubbling into a flask containing deionized water. Afterwards, the extraction cell was opened and the catalyst was recovered (Sample A). The cell was cleaned with acetone, which was then collected and evaporated (Sample B). Samples were analyzed by ICP-OES to determine the amount of precious metal extracted.

$$\text{Extraction conversion (\%)} = \text{E (\%)} = \left(1 - \frac{mass\ of\ Pd\ Sample_A + mass\ of\ Pd\ Sample_B}{mass\ of\ pd\ initial\ catalyst}\right) \times 100$$

The full set of data for the extraction experiments of Cat D is reported in Table S2 and Table S3.

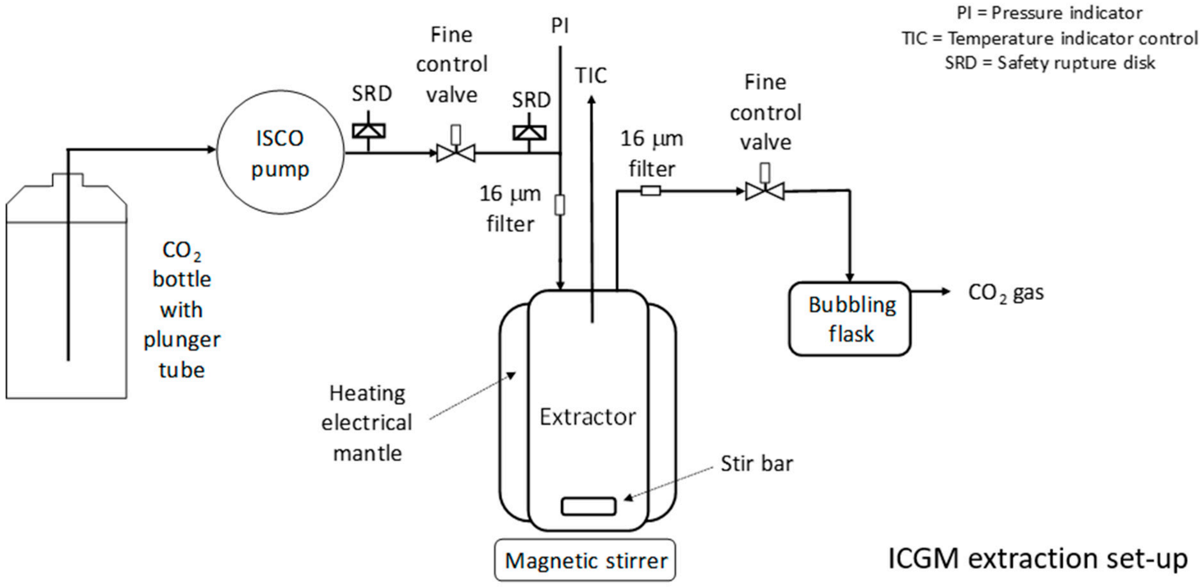

Figure S1. Scheme of the ICGM extraction set-up

Table S1. Sample nomenclature for the ICGM extraction

**Sample Name**	**Sample origin**
Sample A	Catalyst after extraction
Sample B	Washing of extractor with acetone

1.2 Extraction results for Cat D

Table S2. Reactant ratios for extraction experiments performed with Cat D catalysts in supercritical CO_2 at 313 K and 25 MPa[(a)]

Run	Catalyst	(Co)polymer [(b)]	Amount of Polymer [g]	Amount of Catalyst [g]	Additive	Polymer/Pd molar ratio	Complexing group/Pd molar ratio	Additive/Pd molar ratio	Additive/complexing group ratio
**E1**	Cat D	None	-	0.205	-	-	-	-	-
**E2**	Cat D-red	None	-	0.207	-	-	-	-	-
**E3**	Cat D-red-ox	None	-	0.203	-	-	-	-	-
**E4**	Cat D	P(FDA_{11})	1.280	0.203	-	5.735	5.735	-	-
**E5**	Cat D-red	P(FDA_{11})	2.280	0.205	-	10.116	10.116	-	-
**E6**	Cat D-red-ox[(d)]	P(FDA_{11})	1.015	0.206	-	4.482	4.482	-	-
**E7**	Cat D	P($AAEM_{19}$-*co*-FDA_{18})	0.236	0.209	-	0.445	8.450	-	-
**E8**	Cat D	P($AAEM_{19}$-*co*-FDA_{18})	0.248	0.212	TMG	0.461	8.754	10.002	1.143
**E9**	Cat D-red	P($AAEM_{19}$-*co*-FDA_{18})	0.249	0.204	-	0.481	9.134	-	-
**E10**	Cat D-red	P($AAEM_{19}$-*co*-FDA_{18})	0.248	0.203	TMG	0.481	9.142	10.446	1.143
**E11**	Cat D-red-ox[(d)]	P($AAEM_{19}$-*co*-FDA_{18})	0.249	0.211	-	0.465	8.831	-	-
**E12**	Cat D-red-ox[(d)]	P($AAEM_{19}$-*co*-FDA_{18})	0.236	0.207	TMG	0.449	8.532	10.244	1.201
**E13**	Cat D	P($DPPS_7$-*co*-FDA_{18})	0.923	0.203	-	2.086	14.600	-	-
**E14**	Cat D-red	P($DPPS_7$-*co*-FDA_{18})	0.932	0.207	-	2.065	14.457	-	-
**E15**	Cat D-red-ox[(d)]	P($DPPS_7$-*co*-FDA_{18})	0.931	0.204	-	2.093	14.654	-	-
**E16**	Cat D	P($4VP_{20}$-*co*-FDA_{18})	0.259	0.209	-	0.557	11.148	-	-
**E17**	Cat D-red	P($4VP_{20}$-*co*-FDA_{18})	0.251	0.204	-	0.553	11.068	-	-
**E18**	Cat D-red-ox[(d)]	P($4VP_{20}$-*co*-FDA_{18})	0.259	0.208	-	0.560	11.201	-	-

(a) General conditions: $m_{catalyst}$ = 200 mg, $m_{polymer}$ = 250 mg, $m_{CO2, batch\ step}$ = 35 g, $m_{CO2, flushing}$ step = 145 g (1 mg = 10^{-3} g in SI units)

(b) M_n (P(FDA_{11})) = 5850 g/mol, M_n (P($AAEM_{19}$-*co*-FDA_{18})) = 13500 g/mol, M_n (P($DPPS_7$-*co*-FDA_{18})) = 11600 g/mol, M_n (P($4VP_{20}$-*co*-FDA_{18})) = 11800 g/mol

(c) Determined by inductively coupled plasma – optical emission spectrometry (ICP-OES)

(d) For these extractions, 0.02 mL of DIPEA were added to catch any release of HCl from the catalyst. Molar ratio DIPEA/Pd = 3

Table S3. Extraction results and uncertainties of the reaction experiments performed with Cat D catalysts in supercritical CO_2 at 313 K and 25 MPa

Run	Catalyst	(Co)polymer	Initial Pd in Catalyst[(a)] [mg]	Pd in Sample A[(a)] [mg]	Pd in Sample B[(a)] [mg]	Extracted Pd [(b)] (E ± ΔE)[(c)] [%]
**E1**	Cat D	None	4.1170	3.9790	0.1110	0.6 ± 0.6
**E2**	Cat D-red	None	4.4151	4.1990	0.0743	3.2 ± 0.9
**E3**	Cat D-red-ox	None	4.1593	4.0350	0.0598	1.5 ± 0.7
**E4**	Cat D	P(FDA_{11})	4.0768	3.7030	0.0653	7.6 ± 1.4
**E5**	Cat D-red	P(FDA_{11})	4.3724	3.2860	0.2525	19.1 ± 2.9
**E6**	Cat D-red-ox	P(FDA_{11})	4.2207	2.7540	0.3300	26.9 ± 3.8
**E7**	Cat D	P($AAEM_{19}$-*co*-FDA_{18})	4.1973	4.2198	0.1043	0.0 ± 0.1
**E8**	Cat D	P($AAEM_{19}$-*co*-FDA_{18})	4.2576	3.1690	0.6025	11.4 ± 1.9
**E9**	Cat D-red	P($AAEM_{19}$-*co*-FDA_{18})	4.3511	4.0842	0.1553	2.6 ± 0.8
**E10**	Cat D-red	P($AAEM_{19}$-*co*-FDA_{18})	4.3298	3.9420	0.1940	4.5 ± 1.1
**E11**	Cat D-red-ox	P($AAEM_{19}$-*co*-FDA_{18})	4.3232	3.7527	0.1790	9.1 ± 1.6
**E12**	Cat D-red-ox	P($AAEM_{19}$-*co*-FDA_{18})	4.2412	1.9944	0.3575	44.5 ± 6.0
**E13**	Cat D	P($DPPS_7$-*co*-FDA_{18})	4.0768	2.7840	0.2800	24.8 ± 3.6
**E14**	Cat D-red	P($DPPS_7$-*co*-FDA_{18})	4.4151	3.6935	0.0953	14.2 ± 2.3
**E15**	Cat D-red-ox	P($DPPS_7$-*co*-FDA_{18})	4.1798	1.0710	0.2050	69.5 ± 9.2
**E16**	Cat D	P($4VP_{20}$-*co*-FDA_{18})	4.1973	3.3480	0.0195	19.8 ± 2.9
**E17**	Cat D-red	P($4VP_{20}$-*co*-FDA_{18})	4.3511	3.2733	0.0295	24.1 ± 3.5
**E18**	Cat D-red-ox	P($4VP_{20}$-*co*-FDA_{18})	4.2617	1.1111	0.0263	73.3 ± 9.6

(a) Determined by inductively coupled plasma – optical emission spectrometry (ICP-OES
(b) Amount of Pd removed from the catalyst
(c) The uncertainty on the extraction conversion was estimated by applying the following equations:
E = Extraction conversion (%) = $(m_{Pd,initial} - m_{Pd,final}) / m_{Pd,initial}$

$\Delta E/E = [(\Delta m_{Pd,initial} - \Delta m_{Pd,final}) / (m_{Pd,initial} - m_{Pd,final})] + \Delta m_{Pd,initial}/m_{Pd,initial}$

where $m_{Pd,initial}$ refers to the mass of Pd in the supported catalyst before extraction and $m_{Pd,final}$ refers to the total mass of Pd in the supported catalyst (sample A) and left in the extractor (sample B) after extraction

$\Delta m_{Pd,initial}$ (g) = $(0.001 \times m_{Pd,initial} / m_{catalyst,initial}) + 0.06 \times m_{Pd,initial}$ (with weighing accuracy of +/- 1 mg, ICP-OES measurements are accurate within +/-6%)

$\Delta m_{Pd,final}$ (g) = $0.06 \times m_{Pd,initial}$ [(100 - E%) / 100]

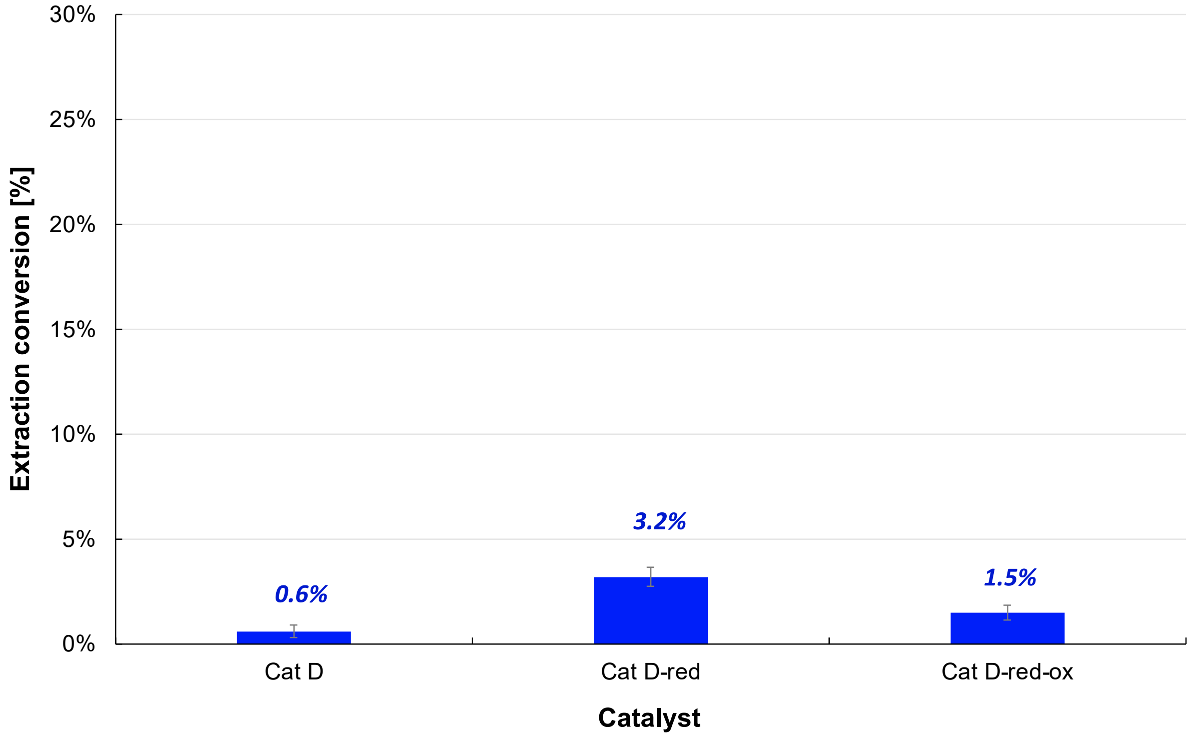

Figure S2. Extraction of palladium from Cat D in the absence of complexing polymer

1.3 Extraction general procedure for Cat A (except E22)

The catalyst and the polymer were placed in the extractor (250 mL stainless steel reactor fitted with a 10 µm sinter (1 µm = 10^{-6} m in SI units)) in the desired amounts. The extractor was closed and the magnetic agitation was set to 250 rpm. The desired temperature (313 K) was adjusted *via* a heating cartridge. Afterwards, an HPLC pump (type: Wadose from Wagner) connected with a Coriolis flow meter (Type: Bronkhorst; M13-AGD-33-O-S; 6–600 g/h CO_2) was used to feed the CO_2 into the extractor (5 g/min) until the desired pressure was obtained (25 MPa) ($m_{CO2} \approx$ 217 g). The extractor outlet was closed by a stop valve (exit valve). After the desired pressure was reached, the CO_2 feed was stopped, and the inlet to the extractor was closed using another stop

valve (inlet valve). Afterwards, the extraction was performed under magnetic stirring at 250 rpm for 90 min (1 min = 60 s in SI units), at 25 MPa and 313 K (batch extraction). The exit valve of the extractor was then opened slowly, so that polymer, Pd, and CO_2 could flow into the separator (stainless steel vessel with sapphire windows), and bubble into an acetone bath. The vent and input of the separator were controlled using two fine valves, one at the extractor and one at the separator outlet. The HPLC pump fed CO_2 to the extractor at about 5 g/min to keep the extractor at 25 MPa and the temperature was kept at 313 K. Approximately 250 to 1500 g of CO_2 was fed through the extractor as a flushing medium to ensure that all the polymer was transported from the extractor to the separator. As the transported polymer could be observed as a continuous polymer precipitation in the acetone bath in the separator (viewed through the sapphire windows), this gave a visual indication of when the flushing process was complete. In the separator, a pressure of 5 MPa and room temperature (293 K) were maintained so that CO_2 in the gas state could leave through a reverse osmosis membrane (Filmtec SW 30, thin film polyamide membrane (PA + PS), Separation limit: NaCl: 99.6%). The Pd and the polymer were collected in the separator acetone bath. After flushing, the pressure in the extraction apparatus was released slowly and the heating was switched off. The supported catalyst was then recovered from the reverse osmosis membrane (Sample e), from the extractor (Sample c), as well as the acetone sample from the separator (Sample a). Afterwards, the separator, including tubing to the extractor, (Sample b) and the extractor itself (Sample d) were washed with acetone separately. For all acetone containing samples, the acetone was evaporated under a fume hood and analyzed by ICP-OES to determine the amount of pd extracted from the catalyst support. The extraction apparatus is presented in Figure S3.

$$\text{Extraction conversion (\%)} = \text{E (\%)} = \frac{m_{Pd,Cat} - m_{Pd,Cat\ after\ extraction}}{m_{Pd,Cat}} \times 100\%$$

The full set of data for the extraction experiments of Cat A is reported in Table S5, Table S6 and Table S7.

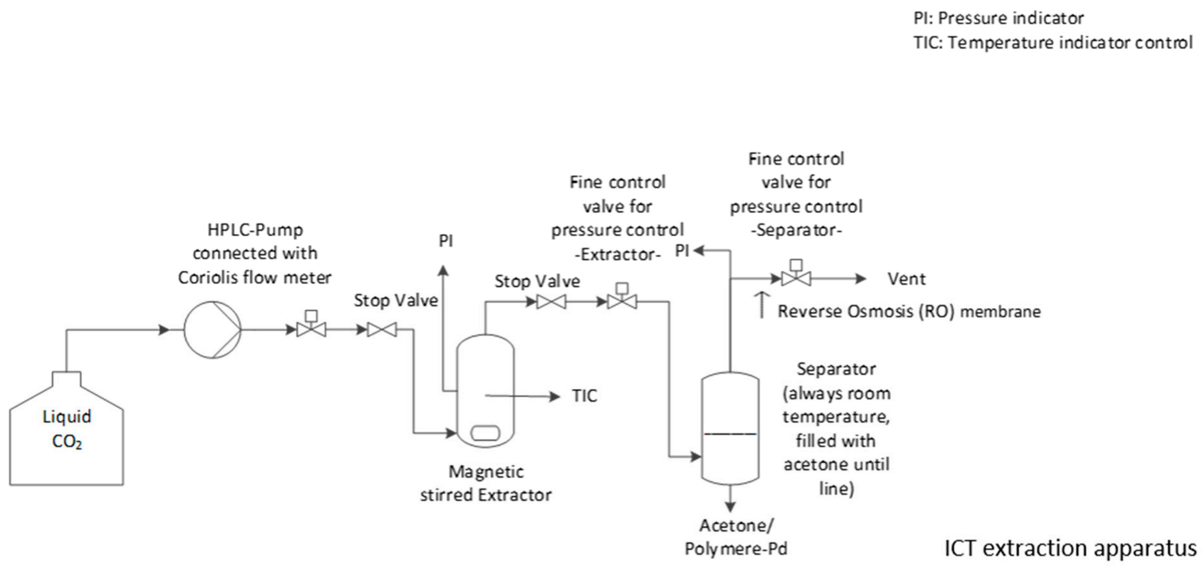

Figure S3. Scheme of the ICT extraction apparatus

Table S4. Sample nomenclature for the ICT extraction

Sample Name	Sample origin
Sample a	Acetone bath separator
Sample b	Washing of separator and pipes with acetone
Sample c	Catalyst after extraction
Sample d	Washing of extractor with acetone

Sample e	Reverse osmosis (RO) membrane

1.4 Extraction results for Cat A

Table S5. Reactant ratios for extraction experiments performed with Cat A catalysts in supercritical CO_2 at 313 K and 25 MPa.[(a)]

Run	Catalyst	(Co)polymer [(b)]	Amount of Polymer [g]	Amount of Catalyst [g]	Polymer/ Pd molar ratio	Complexing group/Pd molar ratio
**E19**[(c)]	Cat A	P(DPPS$_7$-*co*-FDA$_{18}$)	0.526	0.57	1.68	11.75
**E20**	Cat A-red-ox	-	0.000	10.86	-	-
**E21**	Cat A-red-ox	P(DPPS$_8$-*co*-FDA$_{24}$)	1.096	0.28	1.33	48.22
**E22**	Cat A-red-ox	P(4VP$_{20}$-*co*-FDA$_{18}$)	0.135	0.51	0.48	9.65

(a) General conditions: $m_{CO2,\ batch\ step}$ = 215 g, $m_{CO2,\ flushing\ step}$ = 600 g
(b) M_n (P(DPPS$_7$-*co*-FDA$_{18}$)) = 11600 g/mol, M_n (P(DPPS$_8$-*co*-FDA$_{24}$)) = 15300 g/mol, M_n (P(4VP$_{20}$-*co*-FDA$_{18}$)) = 11800 g/mol
(c) $m_{CO2,\ flushing\ step}$ = 1500 g
(d) Determined by inductively coupled plasma – optical emission spectrometry (ICP-OES)

Table S6. Extraction results and uncertainties of the reaction experiments performed with Cat A catalysts in supercritical CO_2 at 313 K and 25 MPa

Run	Catalyst	(Co)polymer [b]	Initial Pd in Catalyst[d] [mg]	Pd in Sample a[a] [mg]	Pd in Sample b[a] [mg]	Pd in Sample c[a] [mg]	Pd in Sample d[a] [mg]	Pd in Sample e[a] [mg]	Extracted Pd [b] ($E \pm \Delta E$)[e] [%]	Pd recovered after extraction[f] ($R \pm \Delta R$) [%]	Pd-Balance[d] ($B \pm \Delta B$)[g] [%]
**E19**[c]	Cat A	P(DPPS$_7$-*co*-FDA$_{18}$)	2.9423	0.0068	0.0005	2.7700	0.0073	0.0004	5.6 ± 0.8	0.26 ± 0.03	94.7 ± 14.6
**E20**	Cat A-red-ox	-	55.5962	0.0039	0.0548	50.6583	1.0700	0.0073	7.0 ± 0.8	0.12 ± 0.01	93.1 ± 11.3
**E21**	Cat A-red-ox	P(DPPS$_8$-*co*-FDA$_{24}$)	1.4532	0.7425	0.2485	0.5452	0.0210	0.0003	61.0 ± 7.9	68.2 ± 9.2	107.2 ± 8.0

(a) Determined by inductively coupled plasma – optical emission spectrometry (ICP-OES
(b) Amount of Pd removed from the catalyst
(c) Amount of Pd recovered after extraction, not including the Pd remaining on the catalyst
(d) Quotient of the amount of Pd found in any part of the extraction apparatus or on the catalyst after extraction, and the amount of pd on the catalyst before extraction
(e) The error on the extraction conversion was estimated by applying the following equations:
E = Extraction conversion (%) = $(m_{Pd,initial} - m_{Pd,final}) / m_{Pd,initial}$
$\Delta E/E = [(\Delta m_{Pd,initial} - \Delta m_{Pd,final}) / (m_{Pd,initial} - m_{Pd,final})] + \Delta m_{Pd,initial}/m_{Pd,initial}$
where $m_{Pd,initial}$ refers to the mass of Pd in the supported catalyst before extraction and $m_{Pd,final}$ refers to the total mass of Pd in the supported catalyst (sample A) and left in the extractor (sample B) after extraction
$\Delta m_{Pd,initial}$ (g) = $(0.001 \times m_{Pd,initial} / m_{catalyst,initial}) + 0.06 \times m_{Pd,initial}$ (with weighing accuracy of +/- 1 mg, ICP-OES measurements are accurate within +/-6%)
$\Delta m_{Pd,final}$ (g) = $0.06 \times m_{Pd,initial}$ [(100 - E%) / 100]
(f) The error on the Pd recovered after extraction was estimated by applying the following equations:
R = Pd recovered after extraction (%) = $(m_{Pd,sample\ a} + m_{Pd,sample\ b} + m_{Pd,sample\ e}) / m_{Pd,initial}$
$\Delta R/R = [(\Delta m_{Pd,initial} - \Delta m_{Pd,recovered}) / (m_{Pd,initial} - m_{Pd,recovered})] + \Delta m_{Pd,initial}/m_{Pd,initial}$
where $m_{Pd,initial}$ refers to the mass of Pd in the supported catalyst before extraction and $m_{Pd,recoveredl}$ refers to the mass of Pd recovered after extraction
$\Delta m_{Pd,recovered}$ (g) = $0.06 \times m_{Pd,initial} \times R\% / 100$
(g) The error on the Pd-Balance was estimated by applying the following equations:

$B = \text{Pd-Balance}\ (\%) = (m_{Pd,sample\ a} + m_{Pd,sample\ b} + m_{Pd,sample\ c} + m_{Pd,sample\ d} + m_{Pd,sample\ e}) / m_{Pd,initial}$

$\Delta B/B = [(\Delta m_{Pd,initial} - \Delta m_{Pd,detected}) / (m_{Pd,initial} - m_{Pd,detected})] + \Delta m_{Pd,initial}/m_{Pd,initial}$

where $m_{Pd,initial}$ refers to the mass of Pd in the supported catalyst before extraction and $m_{Pd,detectedl}$ refers to the mass of Pd detected after extraction anywhere in the system

$\Delta m_{Pd,detected}\ (g) = 0.06 \times m_{Pd,initial} \times B\% / 100$

Table S7. Extraction results and uncertainties of the reaction experiments performed with Cat A catalysts in supercritical CO_2 at 313 K and 25 MPa

Run	Catalyst	(Co)polymer	Initial Pd in Catalyst[(a)] [mg]	Pd in Sample A[(a)] [mg]	Pd in Sample B[(a)] [mg]	Extracted Pd[(b)] ($E \pm \Delta E$)[(c)] [%]
**E22**	Cat A-red-ox	P(4VP_{20}-*co*-FDA_{18})	2.6061	1.440	0.0057	44.5 ± 5.6

(a) Determined by inductively coupled plasma – optical emission spectrometry (ICP-OES)
(b) Amount of Pd removed from the catalyst
(c) The error on the extraction conversion was estimated by applying the following equations:
E = Extraction conversion (%) = $(m_{Pd,initial} – m_{Pd,final}) / m_{Pd,initial}$
$\Delta E/E = [(\Delta m_{Pd,initial} - \Delta m_{Pd,final}) / (m_{Pd,initial} – m_{Pd,final})] + \Delta m_{Pd,initial}/m_{Pd,initial}$
where $m_{Pd,initial}$ refers to the mass of Pd in the supported catalyst before extraction and $m_{Pd,final}$ refers to the total mass of Pd in the supported catalyst (sample A) and left in the extractor (sample B) after extraction
$\Delta m_{Pd,initial}$ (g) = $(0.001 \times m_{Pd,initial} / m_{catalyst,initial}) + 0.06 \times m_{Pd,initial}$ (with weighing accuracy of +/- 1 mg, ICP-OES measurements are accurate within +/-6%)
$\Delta m_{Pd,final}$ (g) = $0.06 \times m_{Pd,initial}$ [(100 - E%) / 100]

2. Catalysts characterization

Catalyst D is a virgin palladium catalyst supported on an aluminosilicate carrier with around 2 wt% of Pd (Figure S4). Macroscopically it consists of small beads of ~0.1 mm (1 mm = 10^{-3} m in SI units) diameter. The average pore size of the aluminosilica support is 21-22 nm (1 nm = 10^{-9} m in SI units) (nitrogen adsorption-desorption isotherms (BET)). The detailed Cat D characterization is reported elsewhere [1] and shown here again, just for reference.

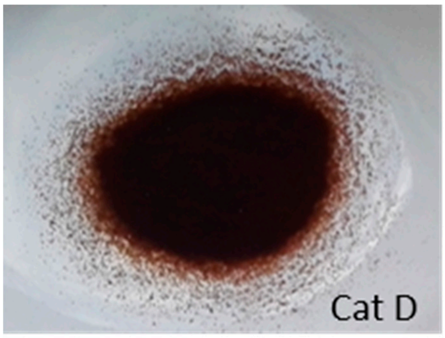

Figure S4. Picture of Cat D

Catalysts Cat D-red and Cat D-red-ox were obtained after a pretreatment (Figure S5).

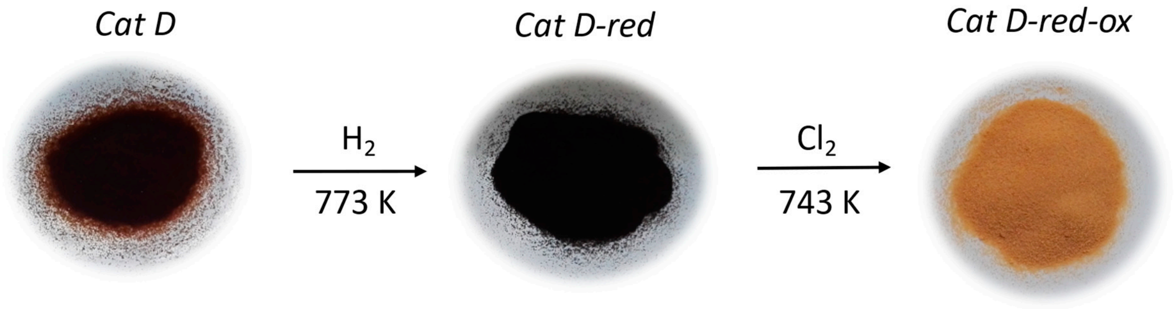

Figure S5. Images of the Pd supported catalysts Cat D, Cat D-red and Cat D-red-ox

Catalyst A is a spent palladium catalyst on an α-alumina carrier with around 0.5 wt% Pd (Figure S6). Macroscopically it consists of large beads of ~5 mm diameter. The Pd nanoparticle size is about 1.9 nm. The specific surface (BET) is quite low with 5.8 m^2/g. Porosity was 0.21 cm^3/g with main (95% of volume) pore diameters in the macroporous range of 105-335 nm. As it is a spent catalyst, Cat A contains 1.3% of carbon. In its delivered form the chemical state of Pd is mainly in the form of PdO. Pretreatment with hydrogen leads to an atomic distribution of 71% Pd^0 and 29% PdO. Further

chlorination leads to a distribution of 11% PdO and 89% Pd-chloride, presumably $PdCl_2$. Note that treated catalyst does not contain carbon anymore.

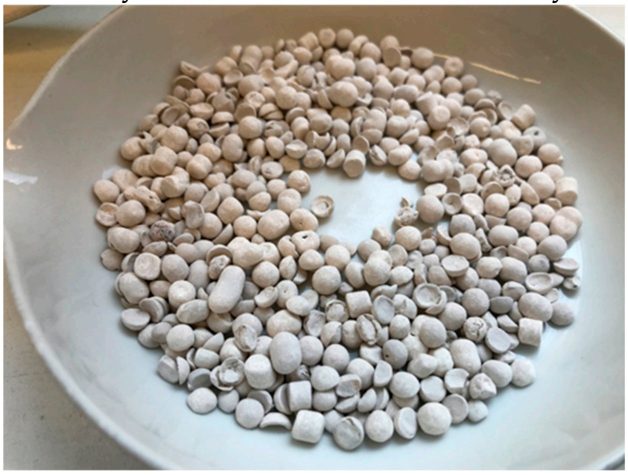

Figure S6. Picture of Cat A

2.1 ICP-OES

2.1.1 Digestion of the supported catalysts

The supported catalysts were ground with a mortar and pestle and passed through a 100 µm (1 µm = 10^{-9} m in SI units) mesh sieve to obtain a fine powder. Then, 0.50 g of the catalyst sample was mixed with 2 mL of 65% nitric acid solution and 6 mL of 37% hydrochloric acid solution (from Merck, Darmstadt, Germany) (12 mL of 37% hydrochloric acid solution for Cat D-red and Cat D-red-ox) in PTFE digestion vessels of a microwave digestion system (Berghof, Eningen, Germany). Then, the digestion program was applied (Table S8). As the supports were not fully digested, the insoluble part was filtered through 0.45 µm cellulose membrane filters. Ultrapure water was added to quote in volumetric flasks of 100 mL. The content of Pd in the obtained solutions was measured by ICP-OES using an Optima 5300DV (Perkin Elmer, Woodbridge, Canada).

Table S8. Digestion program for ICP-OES samples

Step	1	2	3	4
T (K))	373	473	373	298
Time (min))	10	20	5	5

The ICP-OES results for the determination of Pd amount in Cat D, Cat D-red and Cat D-red-ox are reported (Table S9).

Table S9. Pd quantification in Cat D catalysts by ICP-OES

Pd content	Cat D	Cat D-red	Cat D-red-ox

**Xm (mg/kg)**[(a)]	20083	21329	20489
**s (mg/kg)**[(b)]	131	611	363
**RSD (%)**[(c)]	0.65	2.87	1.77

(a) Xm is the average concentration of Pd for n=5 parallel samples
(b) s is the standard deviation
(c) RSD is the relative standard deviation

The ICP-OES results for the determination of Pd amount in Cat A, Cat A-red and Cat A-red-ox are reported (Table S10).

Table S10. Pd quantification in Cat A catalysts by ICP-OES

Pd content	Cat A	Cat A-red	Cat A-red-ox
**Xm (mg/kg)**[(a)]	4954	5177	5080
**s (mg/kg)**[(b)]	299	255	220
**RSD (%)**[(c)]	6.03	4.92	4.33

(a) Xm is the average concentration of Pd for n=5 parallel samples
(b) s is the standard deviation
(c) RSD is the relative standard deviation

2.1.2 Digestion of Sample A (ICGM) and Sample c (ICT) (the catalyst recovered after extraction)

100 - 200 mg sample was weighted in PTFE vessels. Then 15 mL of aqua regia were added and introduced in the microwave oven. Afterwards the digestion program from Table S8 was applied and after digestion, the sample was cooled down to room temperature and filtered on a cellulose filter (circles, diam. 125 mm; from Whatman) using glass funnels, into a fitting volumetric flask. The sample was than diluted to the digested sample volume using ultrapure water. The resulted solution is analyzed by ICP-OES to obtain the mass concentration of Pd in the sample (c_{Pd} [mg/L]).

2.1.3 Digestion of and Sample a, b, d (ICT) (acetone bubble/cleaning solutions)

The whole sample is transferred to PTFE vessels. For this, the bottle sent for analysis containing the sample was washed with aqua regia (in total 14 mL of aqua regia) and introduced in the microwave oven. The digestion program from Table S8 was applied and after digestion, the sample was cooled down to room temperature and filtered on a cellulose filter into a fitting volumetric flask. The sample was diluted to the digested sample volume with ultrapure water. The mass concentration of Pd in the sample was analyzed by ICP-OES (c_{Pd} [mg/L]).

2.1.4 Digestion of Sample e (ICT) (RO-membrane loaded)

The sample (membrane) were introduced to a PTFE vessel of the microwave oven and 14 mL of aqua regia were added. Then the digestion program shown in Table S8 was applied. The digested sample was filtered, after cooling down to room temperature, into a fitting volumetric flask and was diluted to digested sample volume with ultrapure water. Afterwards the mass concentration of Pd in the sample was analyzed by ICP-OES (c_{Pd} [mg/L]).

2.2 SEM-EDX

The SEM-EDX analyses were performed with a ZEISS EVO HD15 coupled with an EDX AZtec (Oxford instrument) apparatus. The catalyst CAT D (2 wt% Pd) was deposited as a powder on a carbon-based, electrically conductive, double-sided adhesive. The samples were prepared by carbon metallization to perform the analysis. This process increments the C % atomic of approximately 2%.

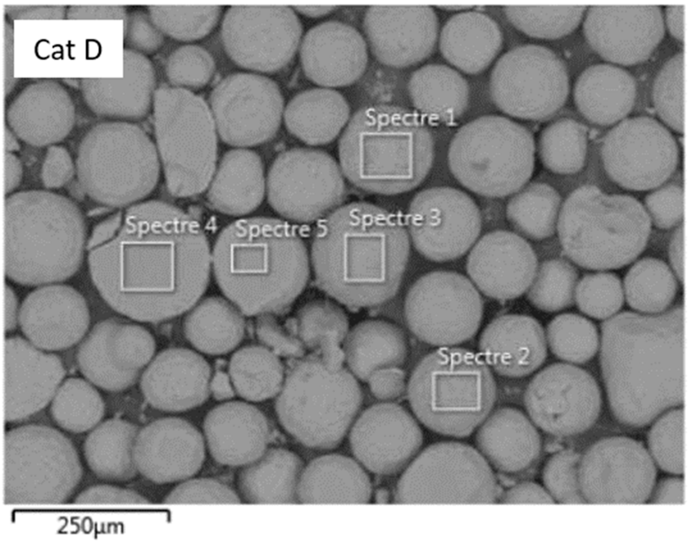

Figure S7. SEM-EDX image of Cat D

Cat D surface

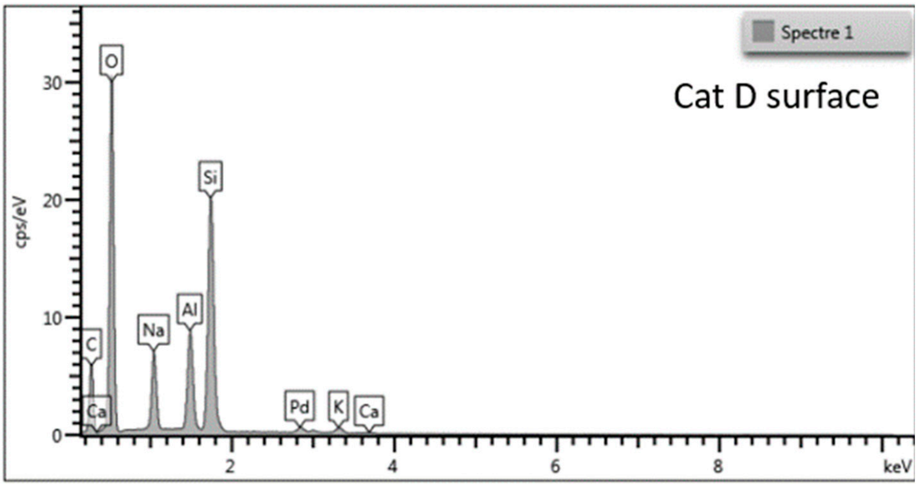

Element	% Mass	% Atomic
C	5.16	8.70
O	42.73	54.35
Na	7.30	6.47
Al	10.33	7.79
Si	28.87	20.92
K	1.77	0.92
Ca	0.36	0.19
Pd	3.47	0.67

Figure S8. EDX and element percentage at the surface of Cat D

Cat D inside

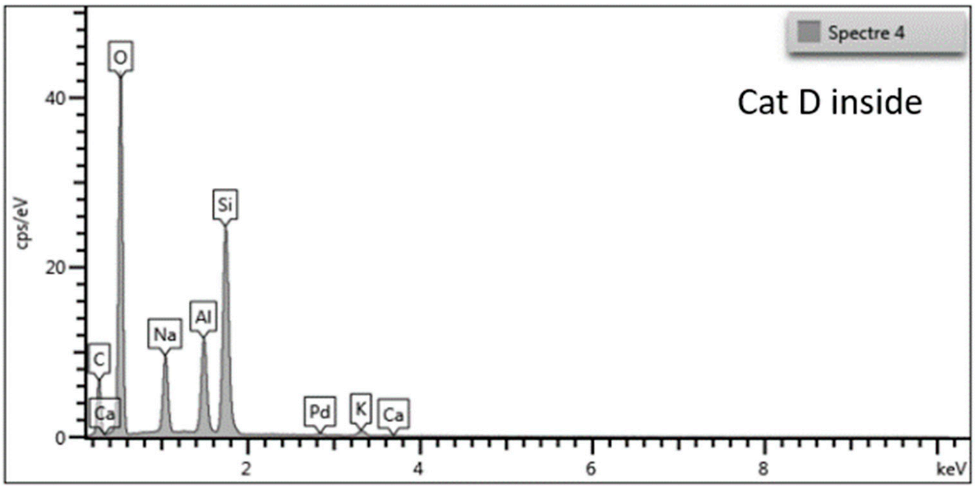

Element	% Mass	% Atomic
C	5.10	8.37

O	46.40	57.14
Na	7.97	6.83
Al	10.32	7.53
Si	27.04	18.97
K	1.56	0.79
Ca	0.26	0.13
Pd	1.37	0.26

Figure S9. EDX and element percentage inside Cat D (fractured bead)

The average size of the catalyst CAT D is 80 micrometers in diameter by SEM-EDX (Figure S7).
From the SEM-EDX studies performed on CAT D, about 71% of the Pd is present on the surface of the catalyst (Figure S8), but the precious metal is also present in the inner part of the support (Figure S9):

$(Pd_{surface}/Al_{surface})/(Pd_{surface}/Al_{surface}+Pd_{inside}/Al_{inside})=(3.47/10.33)/(3.47/10.33+1.37/10.32) = 71.7\ \%$

2.3 Nitrogen adsorption-desorption isotherms (BET), XPS, and TEM

2.3.1 BET analyses

The mesopore size distributions and specific surface areas were determined by nitrogen adsorption-desorption isotherms (BET) using an ASAP-2020 physisorption analyser from Micromeritics (Table S11, Figure S10, Figure S11, Figure S12). The samples were heated at 393 K under reduced pressure (1.3 × 10-3 MPa) for 24 h before the analysis.

Table S11. Specific surface areas and average pore diameters determined by BET for Cat D

Catalyst	Specific surface area	Average pore diameter (adsorption isotherm)
Cat D	122 m^2/g	21 nm
Cat D-red	98 m^2/g	21 nm
Cat D-red-ox	112 m^2/g	22 nm

The pre-treatments of the catalysts did not affect the porosity of the catalyst support, with a constant pore diameter despite the pre-treatments applied on the catalyst, while the specific surface area showed minor changes (Table S11). In addition, the average pore diameter is sufficiently large to allow the fluorinated polymers to enter into the catalyst support.

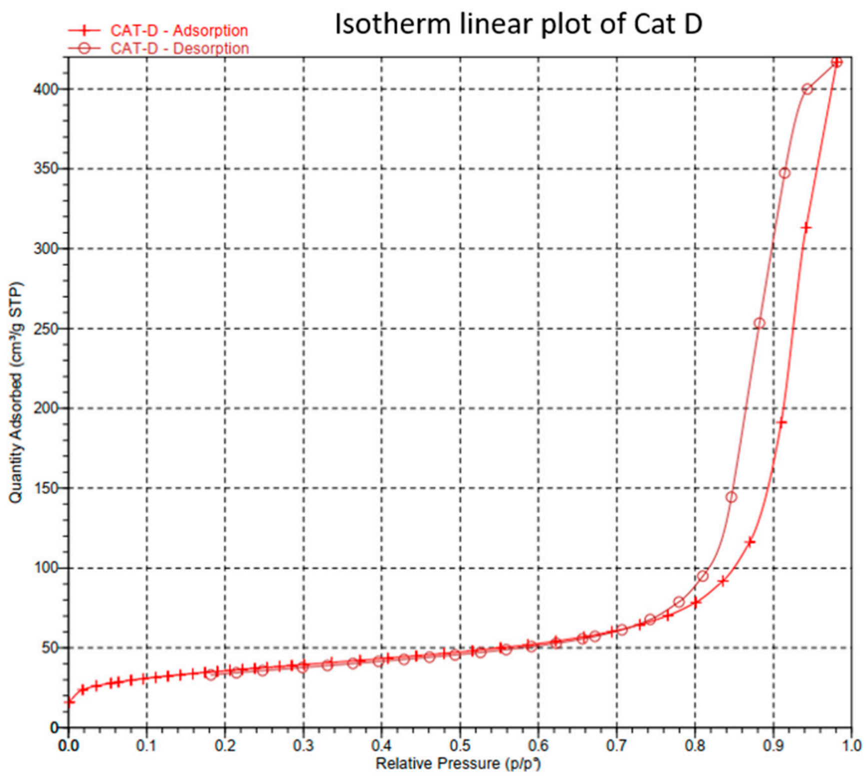

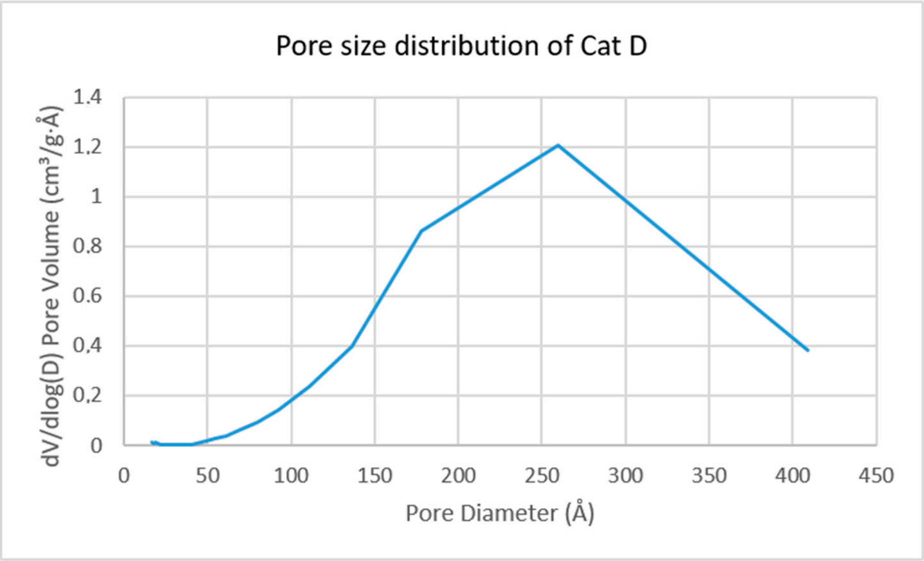

Figure S10. Nitrogen adsorption-desorption isotherms and pore size distribution of Cat D

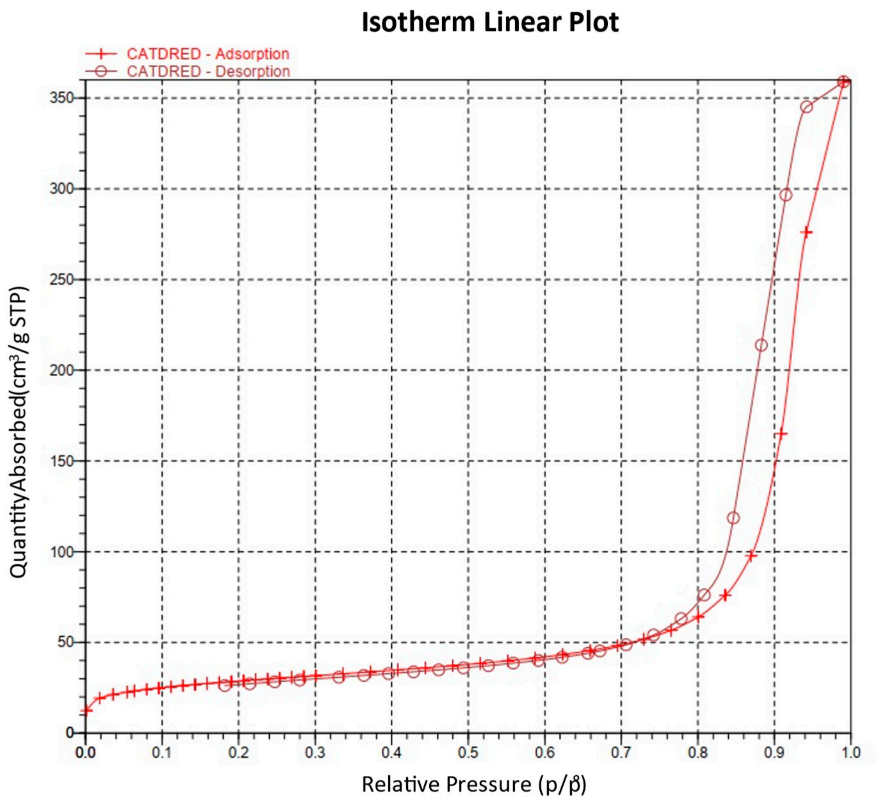

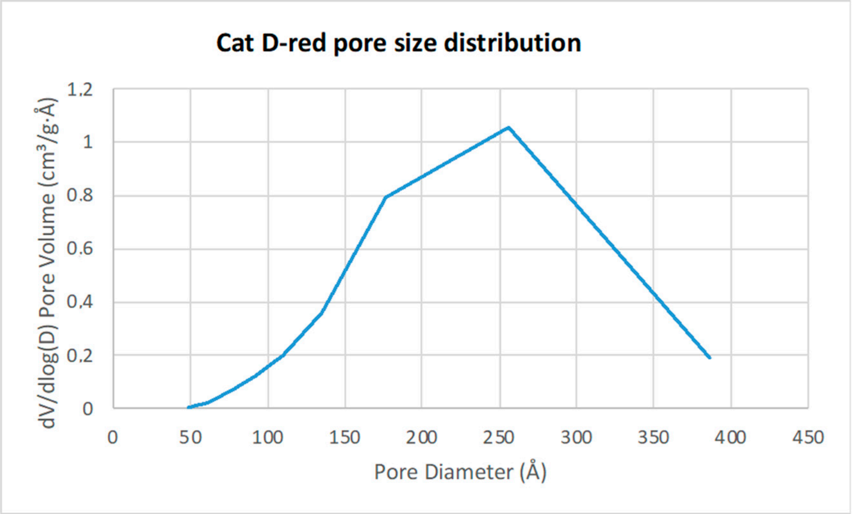

Figure S11. Nitrogen adsorption-desorption isotherms and pore size distribution of Cat D-red

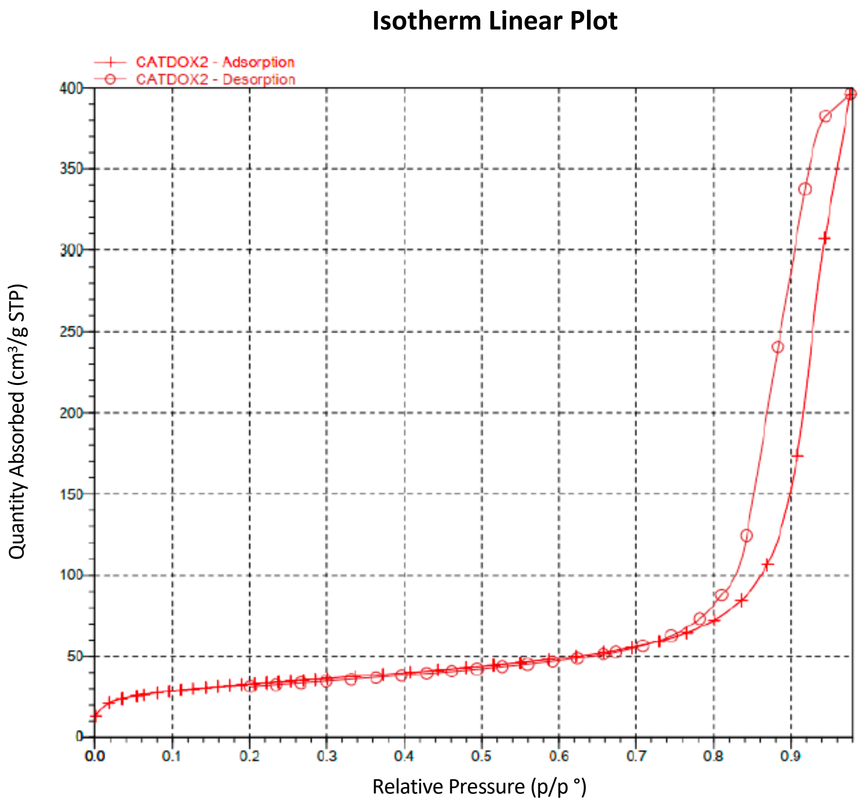

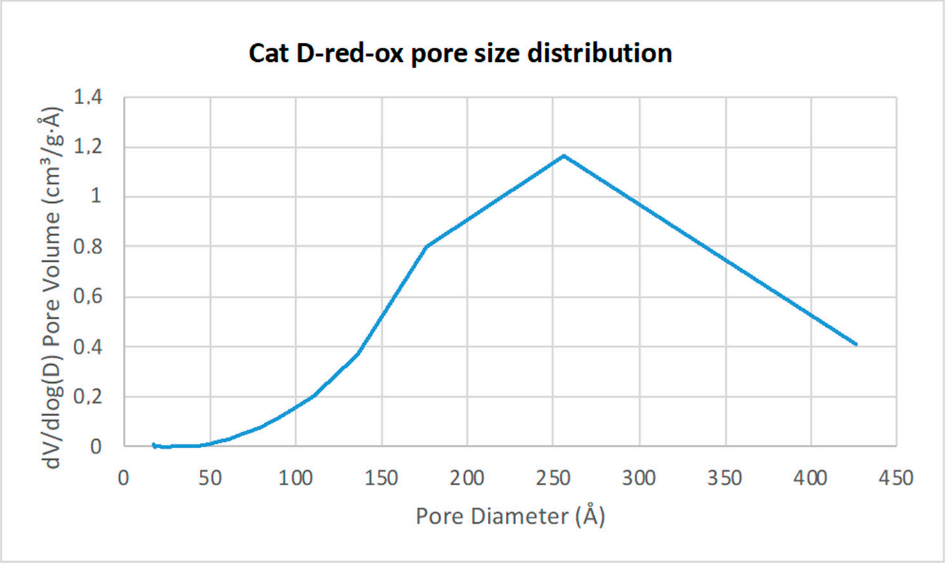

Figure S12. Nitrogen adsorption-desorption isotherms and pore size distribution of Cat D-red-ox

Table S12. Cat A structural characteristics

Parameter	Value	Measurement units
**BET**	5.8	m^2/g
**Particle size**	5	mm

Porosity	0.36	cm^3/g

2.3.2 XPS analyses

XPS measurements were carried out with a THERMO Escalab spectrometer, using focused monochromatic Al Kα radiation (hν = 1486.6 eV). Peaks were recorded with constant pass energy of 20 eV. Charge neutralization was used for all the acquisitions. The pressure in the analysis chamber was around 5×10^{-11} MPa. Short acquisition time spectra were recorded before each experiment to check that the samples did not suffer from degradation during the measurements. The binding energy scale was calibrated using the C 1s peak at 285.0 eV from the hydrocarbon contamination invariably present. The curves fit for core peaks were obtained using a minimum number of components.

Table S13. Elemental composition of Cat D determined by XPS (atomic percentages)

Catalyst	Pd	Al	Si	O	C	Na	K	Cl
Cat D	0.8	9	24	48	12	5	1	-
Cat D-red	0.3	9	25	49	11	4	1	-
Cat D-red-ox	0.5	9	24	45	14	5	1	2

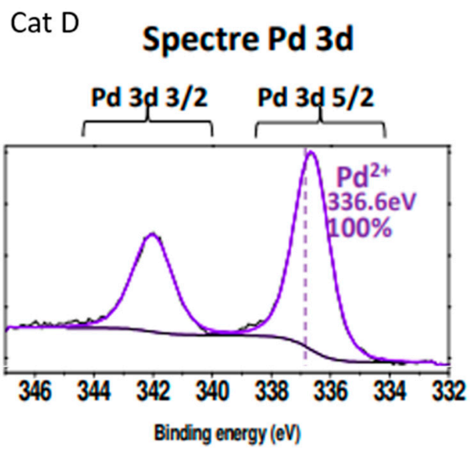

Figure S13. XPS spectrum Pd 3d of Cat D

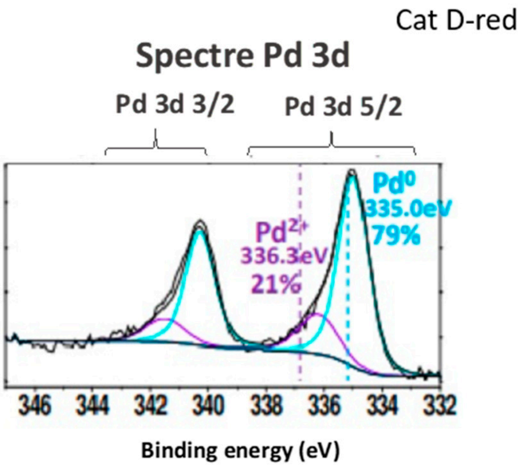

Figure S14. XPS spectrum Pd 3d of Cat D-red

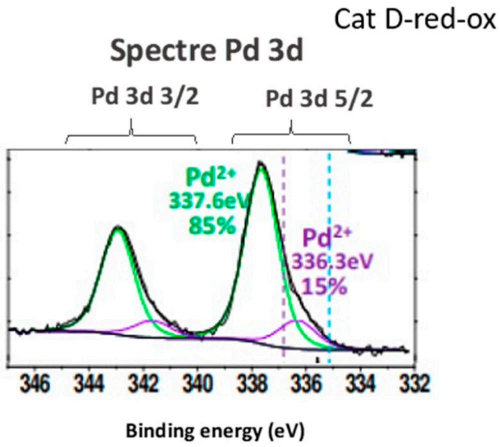

Figure S15. XPS spectrum Pd 3d of Cat D-red-ox

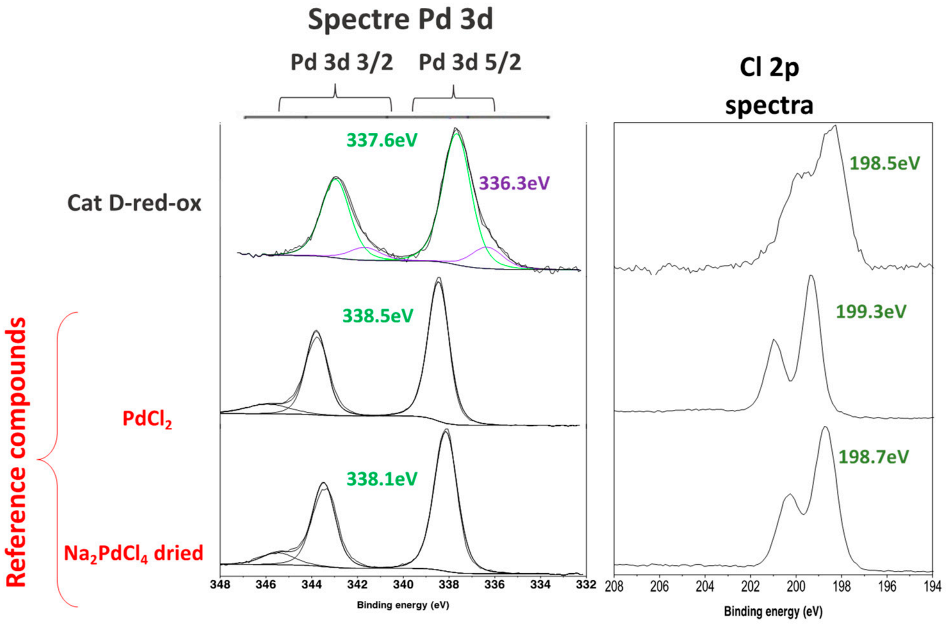

Figure S16. XPS analysis comparison of Cat D-red-ox, $PdCl_2$ and Na_2PdCl_4

The XPS characterization allowed for the study of the oxidation states of the precious metal adsorbed on the aluminosilica support. For the palladium, the Pd 3d spectrum was recorded. For each Pd oxidation state, the Pd 3d spectrum corresponds to a doublet, due to the spin orbit splitting of the d orbital. Hence, for each oxidation degree, the Pd has two peaks named Pd $3d_{5/2}$ and Pd $3d_{3/2}$. The discussion will be focused on the Pd $3d_{5/2}$ peak. For Cat D (Figure S13), the presence of a unique peak at 336.3 eV, typical for Pd(II)O species (100%),[2] was observed. For Cat D-red (Figure S14), the $3d_{5/2}$ spectrum was decomposed into two different peaks: the first one at 336.3 eV, which is still relative to PdO (21%), and a second peak at 335.0 eV, usually assigned to Pd^0 (79%).[2,3] This assignment is consistent with the reduction process applied to Cat D to obtain CAT D-red. Finally, the third catalyst Cat D-red-ox showed a $3d_{5/2}$ spectrum, which was decomposed into two different peaks (Figure S15): the presence of the signal at 336.3 eV was attributed to PdO (15%), while the second peak at 337.6 eV mainly gave information about the environment of the Pd species, which is Pd(II) in a chlorine environment (85%). Considering that the ratio of Pd/Cl is 1:4 (Table S13) and the presence of Na in the catalyst support, this suggests that the new Pd species adsorbed on the catalyst Cat D-red-ox is Na_2PdCl_4. This hypothesis has been confirmed by the XPS analysis of a commercial sample of Na_2PdCl_4 and comparing its XPS spectrum with the one of catalyst Cat D-red-ox. In addition, a commercial sample of $PdCl_2$ was used as another reference. As shown in Figure S16, the peaks of Cl 2p of the Na_2PdCl_4 and of Cat D-red-ox appear at the same binding energy, confirming the presence of Na_2PdCl_4 in the catalyst Cat D-red-ox after oxidation.

Table S14. Elemental composition of Cat A determined by XPS (atomic percentages)

Catalyst	Pd	Al	Si	O	C	Na	Ca	Cl
Cat A	0.5	39		46	15	0.1	0.1	-
Cat A-red	0.4	41		48	10	0.3	0.1	-
Cat A-red-ox	1.2	37		44	13	0.2	-	5

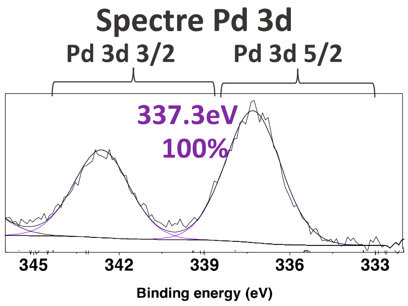

Figure S17. XPS spectrum Pd 3d of Cat A

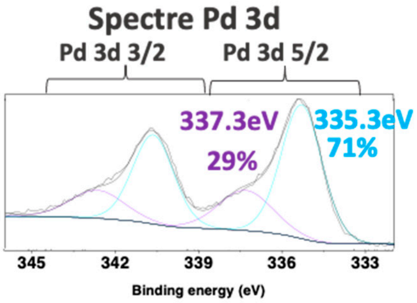

Figure S18. XPS spectrum Pd 3d of Cat A-red

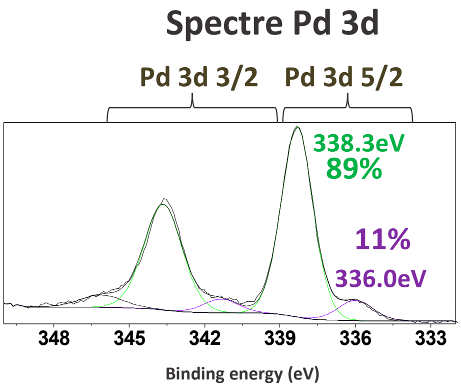

Figure S19. XPS spectrum Pd 3d of Cat A-red-ox

For Cat A (Figure S17), the presence of a unique peak at 337.3 eV, typical for Pd(II)O species (100%) [2], was observed. For Cat A-red (Figure S18), the $3d_{5/2}$ spectrum was

decomposed into two different peaks: the first one at 337.3 eV, which is still relative to PdO (29%), and a second peak at 335.3 eV, usually assigned to Pd^0 (71%) [2,3]. This assignment is consistent with the reduction process applied to Cat A to obtain Cat A red. Finally, the third catalyst Cat A-red-ox showed a $3d_{5/2}$ spectrum, which was decomposed into two different peaks (Figure S19): the presence of the signal at 336.0 eV was attributed to PdO (11%), while the second peak at 338.3 eV mainly gave information about the environment of the Pd species, which is Pd(II) in a chlorine environment (89%), presumably ($PdCl_2$).

2.3.3 TEM analyses

TEM images were obtained with a Jeol 1200EXII transmission electron microscope at an operating voltage of 100 kV with images captured with a Quemesa camera from Olympus Soft Imaging Solutions. Supports were crushed into powder form and embedded into an Embed 812 resin which was then microtomed using an Ultramicrotome Ultracut UCT from Leica Microsystems equipped with a DiATOME ultra diamond knife and placed on a 300-mesh copper grid for TEM analysis.

- *TEM of Cat D*

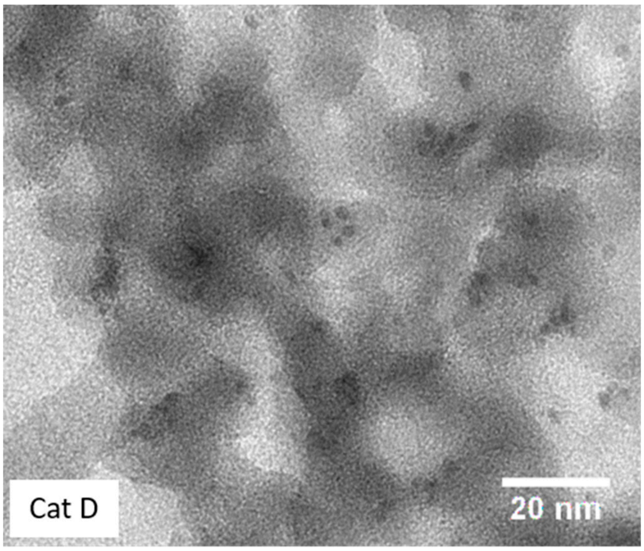

Number-average size (D_n)	
Average [nm]	2.7
Std [nm]	1.1
Median [nm]	2.3
**Mass-average size (D_w)**	
D_w [nm]	4.4
**Polydispersity Index (PDI)**	
D_w/D_n	1.64

Figure S20. TEM and particle size distribution of Cat D

For Cat D (pristine catalyst, 100% PdO), TEM studies showed nanoparticles with an average size of 2.7 nm, with a relatively low dispersity in size (Figure S20).

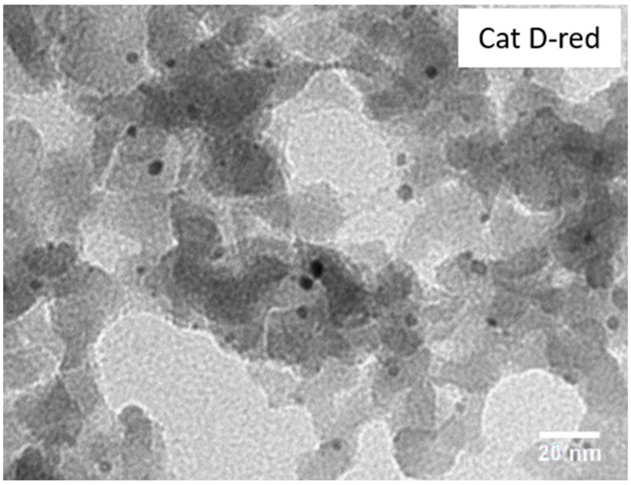

Number-average size (D_n)	
Average [nm]	3.3
Std [nm]	0.5
Median [nm]	3.3
**Mass-average size (D_w)**	
D_w [nm]	3.5
**Polydispersity Index (PDI)**	
D_w/D_n	1.07

Figure S21. TEM and particle size distribution of Cat D-red

For Cat D-red (pre-treated catalyst, 21% PdO, 79% Pd^0), TEM studies showed an increased nanoparticles diameter of 3.3 nm, compared to the pristine catalyst (Figure S21). The increase of the nanoparticles size can be associated with the high temperature used in the reduction treatment (H_2 at 773 K).

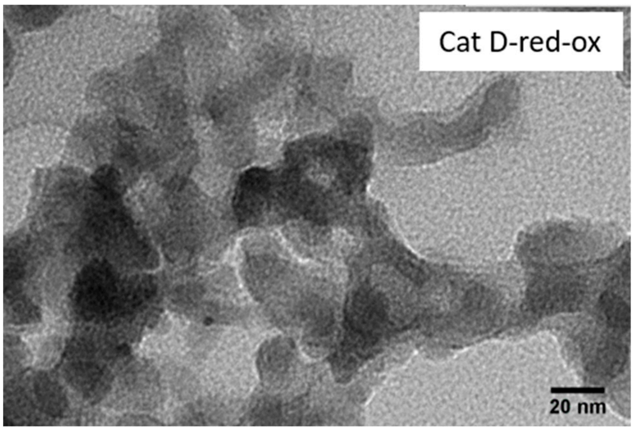

Figure S22. TEM of Cat D-red-ox

For Cat D-red-ox (pre-treated catalyst, 15% PdO, 85% Na_2PdCl_4), TEM analysis displayed a small amount of Pd nanoparticles, related to the remaining 15% of PdO still present on the catalyst (Figure S22). The majority of the precious metal is not detected in TEM, due to the low contrast of Na_2PdCl_4 in TEM.

- *TEM of Cat A*

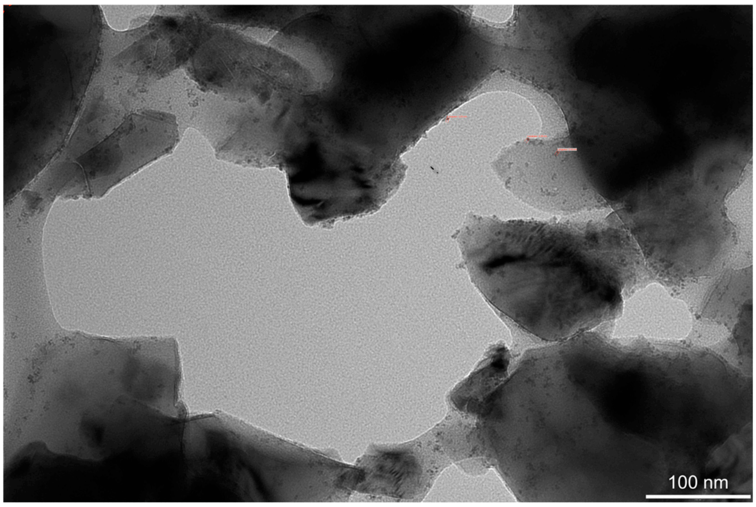

Number-average size (D_n)	
Average [nm]	2.0
Std [nm]	0.5
Median [nm]	1.9
**Mass-average size (D_w)**	
D_w [nm]	2.4
**Polydispersity Index (PDI)**	
D_w/D_n	1.19

Figure S23. TEM and particle size distribution of Cat A

For Cat A (pristine catalyst, 100% PdO), TEM studies showed nanoparticles with an average size of 2.0 nm, with a low dispersity in size (Figure S23).

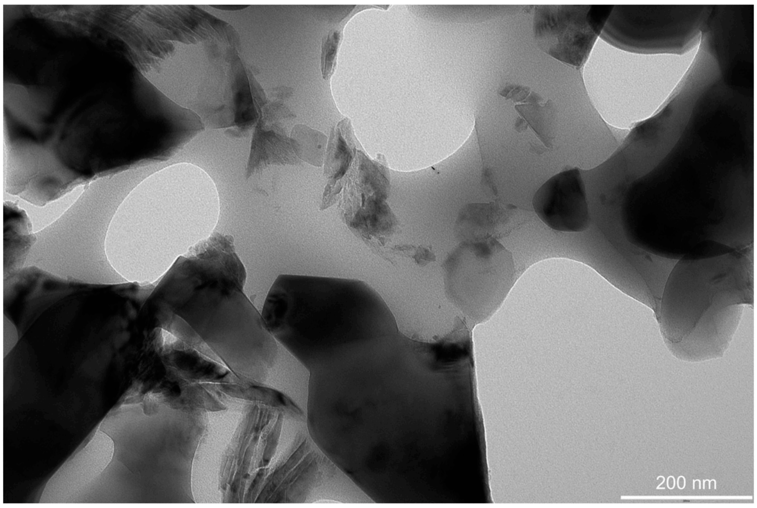

Number-average size (D_n)	
Average [nm]	5.9
Std [nm]	1.4
Median [nm]	5.8
**Mass-average size (D_w)**	
D_w [nm]	6.9
**Polydispersity Index (PDI)**	
D_w/D_n	1.17

Figure S24. TEM and particle size distribution of Cat A-red-ox

For Cat A-red-ox (pretreated catalyst, 11% PdO, 89% $PdCl_2$), TEM analysis displayed a small amount of Pd nanoparticles, related to the remaining 11% of PdO still present on the catalyst (Figure S24). The majority of the precious metal is not detected in TEM, due to the low contrast of $PdCl_2$ in TEM. Besides, TEM studies showed an increase of the nanoparticle diameter to 5.9 nm, compared to Cat A. The increase of the nanoparticles size can be associated with the high temperature used in the reduction treatment (H_2 at 773 K).

3. Polymer synthesis and characterization [4]

3.1 Materials

2,2′-Azobis(2-methylpropionitrile) (AIBN, M = 164.21 g/mol, Fluka, 98%) was purified by recrystallization in methanol and dried under vacuum before use. The chain transfer agent (CTA) ethyl-2-(phenylcarbonothioylthio)propionate (M = 254.36 g/mol) was synthesized and purified as previously reported in the literature [5]. 1,1,2,2-tetrahydroperfluorodecylacrylate (FDA, Mn= 518.17 g/mol, Boc Science, USA, >98%), α, α, α – trifluorotoluene (TFT, Aldrich, >99%), 4-(diphenylphosphino)styrene (DPPS, M= 288.33 g/mol, Aldrich, 97%), 4-vinylpyridine (4VP, M= 105.14 g/mol, Aldrich, 95%), potassium thioacetate (ACROS Organics, M= 114.21 g/mol, 98%) acetoacetoxyethyl methacrylate (AAEM, M= 214.22 g/mol, EASTMAN, 95%), toluene (Aldrich, 99.5%), S-(thiobenzoyl)thioglycolic acid (Aldrich, 99%), sodium hydroxide (NaOH, Fisher), hydrochloric acid (HCl, Aldrich, 37%), sodium sulfate (Aldrich, >99%), ethyl 2-mercaptopropionate (Alfa Aesar, 98%), methanol (Aldrich, 99%), 1,1,2-trichlorotrifluoroethane (CFC-113, Freon 113, Aldrich, 99%), carbon dioxide (CO_2, SFE 5.2, Air Liquide, 99.9%), were used as received unless otherwise indicated.

3.2 (Co)polymers synthesis

- *Synthesis of poly(1,1,2,2-tetrahydroperfluorodecylacrylate) (P(FDA)) homopolymer.*

FDA (40 g, 0.0771 mol), CTA (2.1407 g, 0.0084 mol), AIBN (0.4146 g, 0.0025 mol) and TFT (42 mL) were added in a Schlenk flask. The mixture was stirred magnetically and bubbled for 40 min with N_2. Then, the polymerization was started by heating the Schlenk flask in an oil bath at 338 K. At the end of the reaction, the mixture was let to return to room temperature and it was precipitated in 600 mL of pentane for 3 times from TFT solution and the polymer was dried under vacuum overnight at room temperature. After drying, 32.8 g of polymer were recovered as a fine pink powder (78% yield).

- *Synthesis of poly(1,1,2,2-tetrahydroperfluorodecylacrylate-co-acetoacetoxyethyl methacrylate) (P(AAEM-co-FDA)) copolymer.*

General procedure for copolymerization: FDA (42.0 g, 0.0810 mol), AAEM (18.0 g, 0.0840 mol), CTA (1.5660 g, 0.0061 mol), AIBN (0.3022 g, 0.0018 mol) and TFT (64.6 mL) were added in a Schlenk flask. The mixture was stirred magnetically and bubbled for 40 min with N_2. Then, the polymerization was initiated by heating the Schlenk flask in an oil bath at 338 K. After 96 h, the reaction was let to return to room temperature and it was precipitated in 600 mL of pentane for 3 times from TFT solution and the polymer was dried under vacuum overnight at room temperature. After drying, 26.7 g of polymer were recovered as a fine pink powder (43% yield)

- *Synthesis of poly(1,1,2,2-tetrahydroperfluorodecylacrylate-co-4-(diphenylphosphino)styrene) (P(DPPS-co-FDA)) copolymer.*

The general copolymerization procedure was applied with the following conditions: FDA (42.5 g, 0.0820 mol), DPPS (7.5 g, 0.0260 mol), CTA (1.305 g, 0.0051 mol), AIBN (0.2525g, 0.0015 mol) and TFT (54mL). Reaction time: 96 h. After drying, 31.1 g of polymer were recovered as a fine pink powder (61% yield).

- *Synthesis of poly(1,1,2,2-tetrahydroperfluorodecylacrylate-co-4-vinylpyridine) (P(4VP-co-FDA)) copolymer.*

The general copolymerization procedure was applied with the following conditions: FDA (30.6 g, 0.0590 mol), 4VP (5.4 g, 0.0513 mol), CTA (0.936 g, 0.0036 mol), AIBN (0.180 g, 0.0011 mol) and toluene (36 mL). Reaction time: 120 h. After drying, 21.7 g of polymer were recovered as a fine pink powder (59% yield).

3.3 (Co)polymer characterization

Polymer compositions were determined by ^{1}H-NMR spectroscopy on a Bruker Avance 400 MHz spectrometer at room temperature. The spectra were recorded by dissolving 10 mg of sample in 0.5 mL of $CDCl_3$ (4VP-based copolymers), acetone-d_6 (AAEM copolymers), CFC-113 with C_6D_6 capillaries (other fluorinated (co)polymers).

3.4 (Co)polymers synthesized

Table S15. Synthesis by RAFT polymerization of P(FDA) and gradients complexing copolymers

**(Co)polymer** (a)	$\mathbf{M_{n,targeted}}$ (c) **[g/mol]**	$\mathbf{DP_{complexing\ mono}}$ (d)	$\mathbf{DP_{FDA}}$ (d)	$\mathbf{M_{n,NMR}}$ (d) **[g/mol]**	$\mathbf{f_{complexing\ mono}}$ (e) **[wt%]**	$\mathbf{f_{FDA}}$ (f) **[wt%]**
P(FDA)	5020	0	10.8	5850	0	100
P(AAEM-*co*-FDA)	10090	18.9	17.7	13480	30.6	69.4
P(DPPS-*co*-FDA)	10060	7.5	17.8	11640	19.0	81.0
P(4VP-*co*-FDA) (b)	10250	19.8	18.3	11820	18.0	82.0

(a) (Co)polymerization of FDA and complexing monomers by RAFT in TFT at 338 K with molar ratio AIBN/CTA = 0.3
(b) Use of toluene instead of TFT as reaction solvent.
(c) $M_{n,targeted} = ((m_{FDA} + m_{complexing\ monomer})/n_{CTA}) + M_{CTA}$, where m_{FDA} and $m_{complexing}$ monomer are the mass of FDA and complexing monomers, n_{CTA} is the moles of CTA necessary for the polymerization and M_{CTA} is the molecular weight of the chain end groups (254.36 g/mol).
(d) Determined by ^{1}H-NMR peak intensity ratio.
(e) $f_{complexing\ mono}$ = fraction of complexing monomer unit = ratio of the weight of complexing monomer units with respect to the total weight of complexing monomer and FDA, determined by ^{1}H-NMR peak intensity ratio.
(f) f_{FDA} = fraction of FDA monomer unit = ratio of the weight of FDA units with respect to the total weight of complexing monomer and FDA, determined by ^{1}H-NMR peak intensity ratio.

- *P(FDA₁₁)* (used in E4, E5, E6)

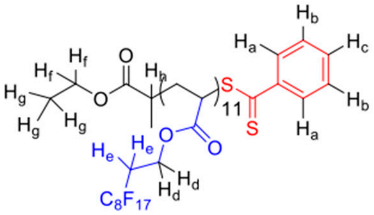

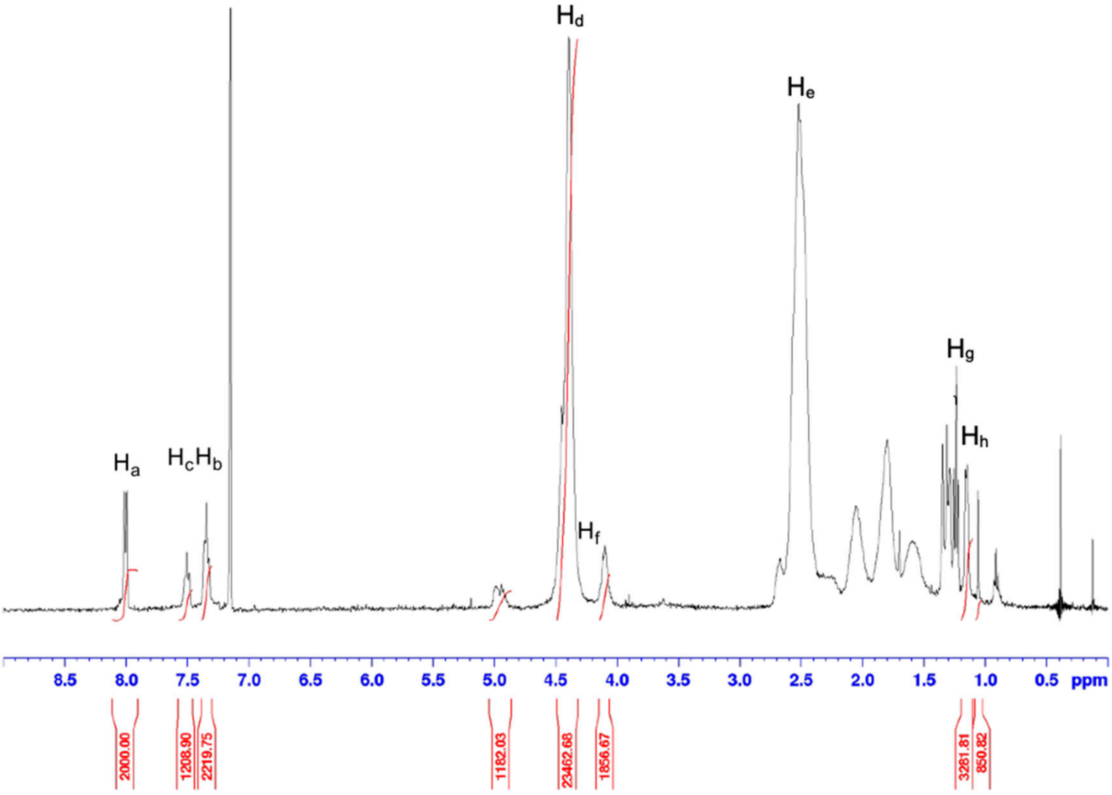

Figure S25. ^{1}H-NMR spectrum (400 MHz, CFC-113 + C_6D_6 capillaries) of P(FDA) after precipitation

The degree of polymerization (DP_{FDA}) of the monomer unit (FDA) was calculated based on the following formula where H_i corresponds to the integral of the protons i in the ^{1}H-NMR spectrum (figure S25):

$$DP_{FDA} = \frac{Hd/_2}{(Ha + Hb + Hc)/_5} = 10.8$$

where H_a, H_b, H_c and H_d correspond to the integrals associated to the protons a, b, c and d from CTA aromatic end-group and FDA.

$$M_{n,precipitated,P(FDA)}\ (g/mol) = DP_{FDA} \times M_{FDA} + M_{CTA} = 5850\ g/mol$$

where M_{FDA} and M_{CTA} refer to the molecular weight of FDA (518.17 g/mol) and CTA (254.36 g/mol).

- *P(AAEM19-co-FDA18)* (used in E7, E8, E9, E10, E11, E12)

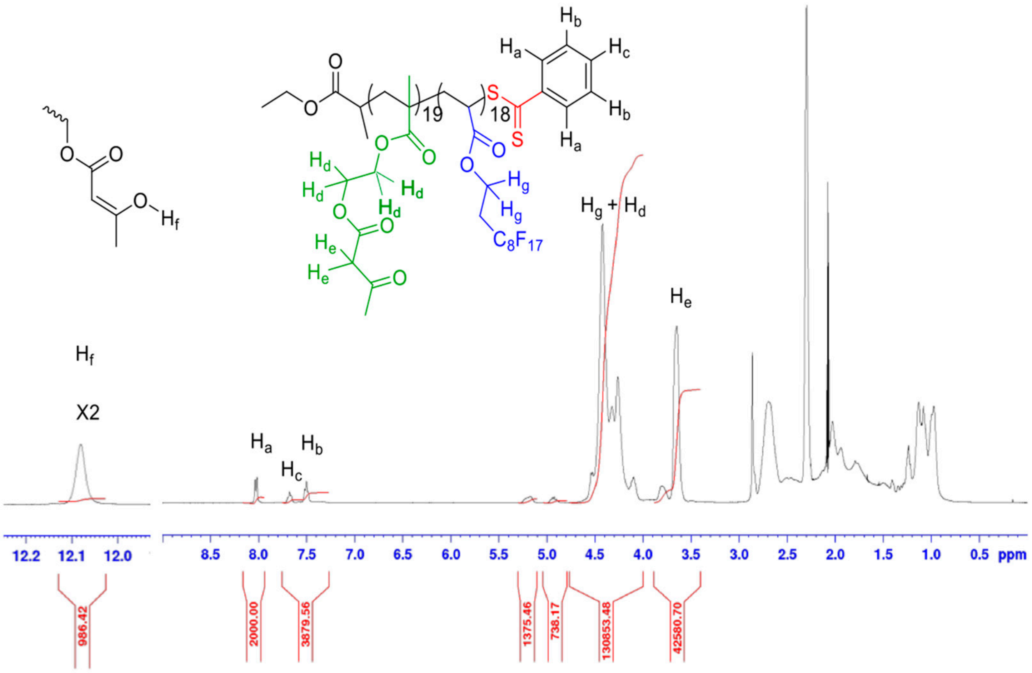

Figure S26. ^{1}H-NMR spectrum (400 MHz, acetone-d6) of P(AAEM-*co*-FDA) after precipitation

The calculation of the degrees of polymerization and copolymer molecular weight have been done taking into account both ketone and enol forms of the AAEM monomer units as illustrated in the ^{1}H-NMR spectrum with 4.5 mol% of the enolic conformation (Figure S26). The calculation was based on the following formula where H_f corresponds to the integral of the protons f (Figure S26).

$$DP_{AAEM} = \frac{\left(\left(Hf + {}^{He}/_{2}\right)\right)}{{}^{(Ha + Hb + Hc)}/_{5}} = 18.9$$

$$DP_{FDA} = \frac{{}^{\left((Hg + Hd) - 4\left(Hf + {}^{He}/_{2}\right)\right)}/_{2}}{{}^{(Ha + Hb + Hc)}/_{5}} = 17.7$$

$$M_{n,precipitated\ P(AAEM-co-FDA)}\ (g/mol) = DP_{AAEM} * M_{AAEM} + DP_{FDA} * M_{FDA} + M_{CTA} = 13480\ g/mol$$

where M_{AAEM}, M_{FDA} and M_{CTA} refer to the molecular weight of AAEM (214.22 g/mol), FDA (518.17 g/mol) and CTA (254.36 g/mol).

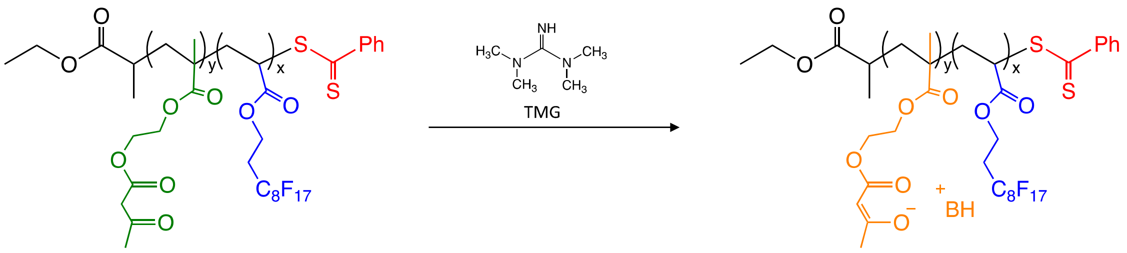

Figure S27. Activation of acetoacetoxy complexing group in the presence of TMG to form the enolate group

- *P(DPPS₇-co-FDA₁₈)* (used in E13, E14, E15, E19)

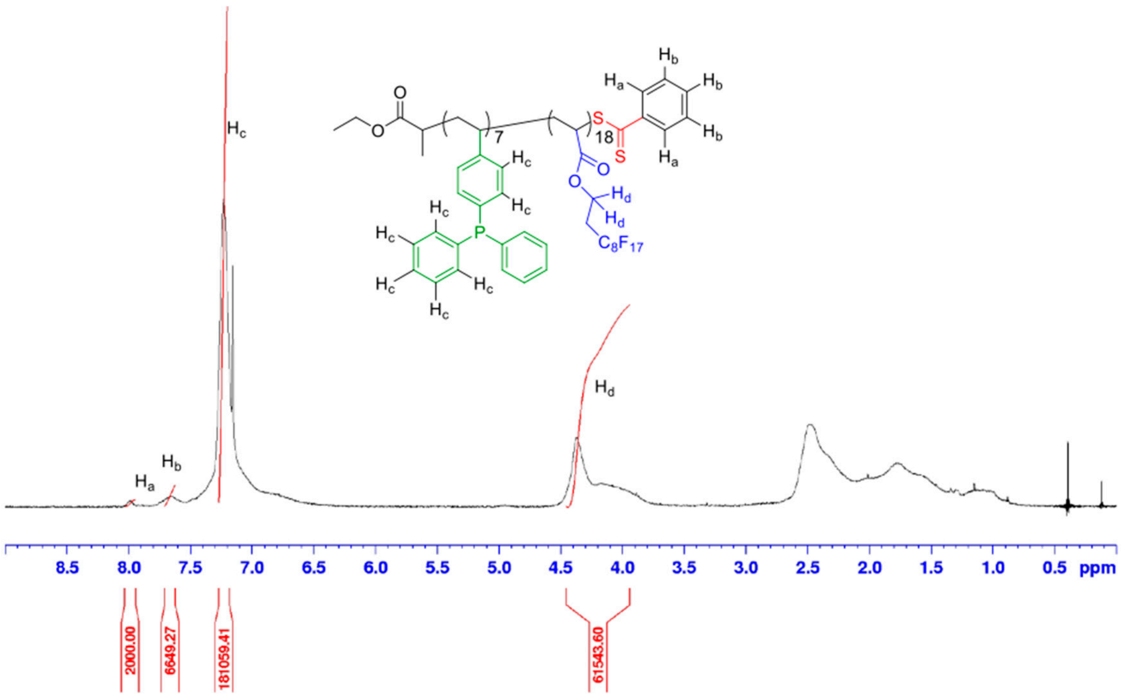

Figure S28. ^{1}H-NMR spectrum (400 MHz, CFC-113 + C_6D_6 capillaries) of P(DPPS-*co*-FDA) after precipitation.

The degrees of polymerization (DP_{FDA} and DP_{DPPS}) of the two different units and the molecular weight of the copolymer were estimated based on the following formula where H_i corresponds to the integral of the protons i in the ^{1}H-NMR spectrum (Figure S28):

$$DP_{DPPS} = \frac{Hc/_{14}}{(Ha + Hb)/_{5}} = 7.5$$

$$DP_{FDA} = \frac{Hd/_{2}}{(Ha + Hb)/_{5}} = 17.8$$

$$M_{n,precipitated\ P(DPPS-co-FDA)}\ (g/mol) = DP_{DPPS} \times M_{DPPS} + DP_{FDA} \times M_{FDA} + M_{CTA} = 11640\ g/$$

where M_{DPPS}, M_{FDA} and M_{CTA} refer to the molecular weight of DPPS (288.32 g/mol), FDA (518.17 g/mol) and CTA (254.36 g/mol).

- *P(4VP$_{20}$-co-FDA$_{18}$)* (used in E16, E17, E18, E22)

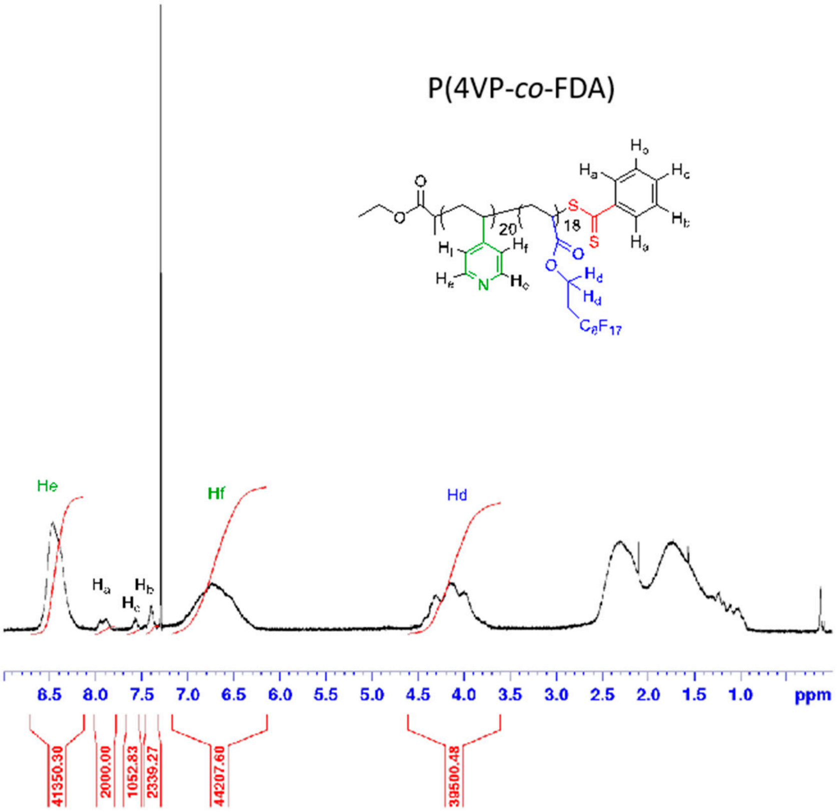

Figure S29. ^{1}H-NMR spectrum (400 MHz, $CDCl_3$) of P(4VP-*co*-FDA) after precipitation.

The degrees of polymerization (DP_{FDA} and DP_{4VP}) of the two different units and the molecular weight of the copolymer were estimated based on the following formula

where H_i corresponds to the integral of the protons i in the ^{1}H-NMR spectrum (Figure S29).

$$DP_{4VP} = \frac{\left(\frac{(He + Hf)}{4}\right)}{\frac{(Ha + Hb + Hc)}{5}} = 19.8$$

$$DP_{FDA} = \frac{\frac{Hd}{2}}{\frac{(Ha + Hb + Hc)}{5}} = 18.3$$

$$M_{n,\text{precipitated P(4VP}-co-\text{FDA)}}\ (g/mol) = DP_{4VP} \times M_{4VP} + DP_{FDA} \times M_{FDA} + M_{CTA} = 11820\ g/mol$$

where M_{4VP}, M_{FDA} and M_{CTA} refer to the molecular weight of 4VP (105.14 g/mol), FDA (518.17 g/mol) and CTA (254.36 g/mol).

3.5 Cloud point curves of the polymers in dense CO_2

Cloud point measurements were carried out in a high pressure, variable volume view cell equipped with a sapphire window on the end for visual observations. The cell was equipped with a pressure transducer and an internal thermocouple. It was thermostated by a water/isopropanol alcohol mixture delivered by a Lauda RE206 circulating pump. CO_2 is delivered by an ISCO 260D automatic syringe pump. An amount of 55 mg of polymer was weighed and transferred to the cell along with a clean stirring bar at a starting volume of 6.40 mL. Subsequently, the cell was fed with CO_2 at about 298 K and 10.9 MPa. Then, the cell was heated to 338 K (taking care to adjust the volume of the cell in order to stay below a pressure of 35 MPa; safety rupture disk at 50 MPa). Cloud points (one-phase/two-phase transition) were obtained by decreasing the pressure of the cell by increasing the cell volume through a hand-driven piston after 15 min of stirring at a given temperature. The cell was cooled by steps of 278 K down to 298 K. The uncertainty of the cloud point pressure was 0.5 MPa.

The cloud points of the (co)polymers used in this study are presented (Figure S30).

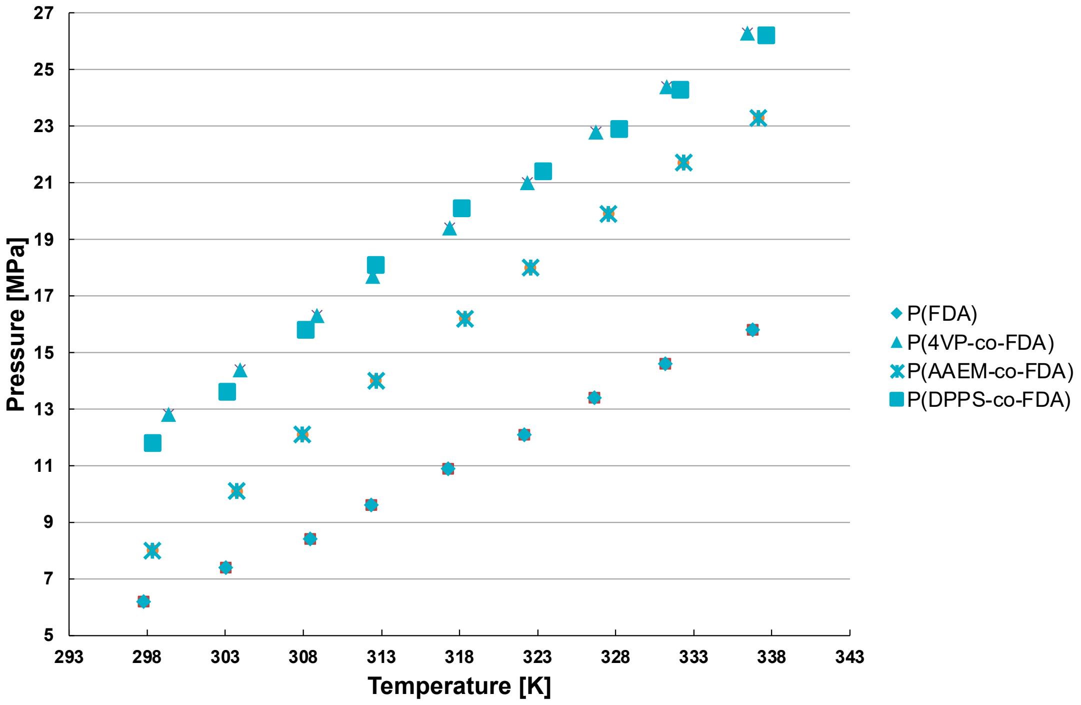

Figure S30. Cloud point curves in dense CO_2 of the (co)polymers used in this study (at a polymer concentration of ca. 1 wt% of polymer relative to CO_2)

4. References

[1] A. Ruiu, B. Bauer-Siebenlist, M. Senila, T. Jänisch, D. Foix, K. Seaudeau-Pirouley, P. Lacroix-Desmazes, Promising Polymer-Assisted Extraction of Palladium from Supported Catalysts in Supercritical Carbon Dioxide. *J. CO2 Util.* **2020**, *41*, 101232.

[2] K. Otto, L.P. Haack, J.E. deVries, Identification of two types of oxidized palladium on γ-alumina by X-ray photoelectron spectroscopy, *Appl. Catal. B, Environ.* **1992**, *1*, 1–12.

[3] M.C. Militello, S.J. Simko, Elemental Palladium by XPS, *Surf. Sci. Spectra*. **1994**, *3*, 387–394.

[4] A. Ruiu, C. Bouilhac, O. Gimello, K. Seaudeau-Pirouley, M. Senila, T. Jänisch, P. Lacroix-Desmazes Synthesis and Phase Behavior of a Platformof CO2-Soluble Functional Gradient Copolymers Bearing Metal-Complexing Units *Polymers* **2022**, *14*, 2698

[5] R. Severac, P. Lacroix-Desmazes, B. Boutevin, Reversible addition-fragmentation chain-transfer (RAFT) copolymerization of vinylidene chloride and methyl acrylate, *Polym. Int*. **2002**, *51*, 1117–1122
